# The response regulator FpsR controls the flagella–pili transition and mucosal colonization in *Ligilactobacillus ruminis*

**DOI:** 10.1080/19490976.2025.2596807

**Published:** 2025-12-04

**Authors:** Aya Misaki, Shunya Suzuki, Shintaro Maeno, Akihito Endo, Yasuko Sasaki, Gen Enomoto, Kenji Yokota, Akinobu Kajikawa

**Affiliations:** a Department of Agricultural Chemistry, Tokyo University of Agriculture, Tokyo, Japan; b Biomanufacturing and Process Research Center, National Institute of Advanced Industrial Science and Technology, Ibaraki, Japan; c Research Center for Thermotolerant Microbial Resources, Yamaguchi University, Yamaguchi, Japan; d Department of Nutritional Science and Food Safety, Tokyo University of Agriculture, Tokyo, Japan; e Department of Agricultural Chemistry, Meiji University, Kanagawa, Japan

**Keywords:** *Ligilactobacillus ruminis*, flagella, motility, pili, gut colonization

## Abstract

*Ligilactobacillus ruminis* is a flagellated lactic acid bacterium found in the intestines of various mammals, including humans. Although this species harbors a complete flagellar gene cluster, flagella formation has not been observed in human-derived strains, and the underlying regulatory mechanisms remain unknown. Here, we isolated a motility-acquired mutant of *L. ruminis* ATCC 25644 that exhibited full flagellation and a measurable chemotactic response under acidic conditions (pH 3.0). Whole-genome sequencing revealed a ~35 kb deletion encompassing multiple regulatory genes. Functional complementation identified a single response regulator, designated FpsR (flagellation–piliation switchover regulator), as a central switch that suppresses flagella formation while promoting pilus expression. The motility-acquired mutant displayed reduced pilus production, diminished adhesion to murine intestinal mucus and fibronectin, and increased susceptibility to acid (pH 3.0) and bile (0.25–0.5%), resulting in a complete loss of intestinal colonization in a murine model. Furthermore, while flagellin from the motile strain activated TLR5 and induced proinflammatory responses comparable to those of pathogenic bacteria, no such inflammation was observed in vivo, likely due to the strain’s colonization failure. These findings reveal FpsR as a previously unrecognized genetic mechanism that coordinates motility and mucosal colonization in a human commensal bacterium and provide insight into how flagella are regulated and silenced in the gut environment to support host–microbe symbiosis.

## Introduction

The gastrointestinal tract of animals harbors a dense and diverse community of microbes that profoundly influences host physiology, metabolism, and immunity. Colonization of the intestinal mucosa by individual bacterial species is achieved through species-specific mechanisms that have evolved to ensure persistence in the dynamic and competitive gut environment. Among commensal bacteria such as *Bifidobacteria* and lactic acid bacteria, the formation of pili has emerged as a key strategy for mucosal adherence. For example, the pili of *Bifidobacterium breve* UCC2003 have been shown to be essential for colonization in the gut, and the pilin proteins of *Bifidobacterium bifidum* PRL2010 are suggested to promote adhesion to both epithelial cells and extracellular matrix components ^1,2^. In lactic acid bacteria, pili from *Lacticaseibacillus rhamnosus* GG have been shown to mediate adhesion, biofilm formation, and interaction with host tissues.^3-5^

While pili facilitate mucosal colonization, flagella confer motility. Flagella-mediated motility and chemotaxis are well-characterized virulence factors in pathogens such as *Salmonella*, *Vibrio*, and *Helicobacter*, where they contribute to mucosal penetration, immune evasion, and host invasion.^6-11^ However, the presence of flagella and motility is rare among commensal gut bacteria and thus the biological significance of flagella in non-pathogenic, symbiotic gut bacteria remains poorly understood. Over the past decade, reports on non-pathogenic motile bacteria in the animal gut have emerged. For instance, *Ligilactobacillus agilis*, a motile lactic acid bacterium found in various animals, displays chemotaxis toward gut-derived compounds while exhibiting minimal proinflammatory activity via its flagellin.^12-15^ Similarly, FlaB (flagellin) from *Roseburia hominis*, a common human gut commensal, has been described as immunologically attenuated,^16^ supporting the idea of functional divergence between commensal and pathogenic flagellins.

*Ligilactobacillus ruminis*is a commensal lactic acid bacterium frequently isolated from the intestines of various mammals, including humans. It is considered beneficial to the host, with reported roles in antiviral defense and mucosal immune stimulation.^17-19^ Both flagella and pili formation have been described in a strain-dependent manner.^5^^,^^20-22^ Among lactic acid bacteria inhabiting the human gut, *L. ruminis* is the most prevalent species, according to a large-scale metagenomic study,^23^ and it appears to be the only known lactobacillus in the human intestine that possesses a complete set of flagella-related genes. *L. ruminis* ATCC 25644 is the only publicly preserved human gut-derived strain of this species. Pili formation has previously been reported in this strain, and it is suggested to be involved in adhesion to the extracellular matrix and intestinal epithelial cells.^5^ Although it retains a full set of motility genes with 97% sequence identity to those of the bovine-derived motile strain *L. ruminis* ATCC 27782, neither flagella formation nor full motility has been observed in ATCC 25644 or other human-derived isolates.^24^

Although uncommon, commensal flagellated bacteria have drawn growing interest due to their potential roles in host–microbe interactions, yet the molecular basis governing their motility and colonization remains largely elusive. In this study, we isolated a spontaneous motility-acquired mutant from *L. ruminis* ATCC 25644 and characterized its phenotypic and immunological properties. Our analyzes revealed that flagella formation confers chemotactic behavior and proinflammatory potential but compromises colonization capacity by suppressing pili formation. Further genomic profiling and genetic modification identified a transcriptional regulator that inversely controls flagella and pili expression. These findings provide new insight into the genetic regulation of surface structures in gut commensals and suggest that *L. ruminis* dynamically modulates its motile and adhesive states to balance host interaction, immune evasion, and niche persistence.

## Materials and methods

### Bacterial strains and culture conditions

The bacterial strains and plasmids used in this study are listed in Table 1. *Ligilactobacillus ruminis* strains were cultured at 37 °C in either liquid or solid MRS medium (Difco, BD), with agar concentrations of 0.2−0.5% or 1.5% as appropriate. Due to its high oxygen sensitivity, cultivation was performed either in an anaerobic chamber (BAKER; 75% N₂, 20% CO₂, 5% H₂) or in sealed glass vials. Where necessary, erythromycin (5 µg/mL), streptomycin (50–100 µg/mL), or kanamycin (25 µg/mL) was added to the medium. *Lactococcus lactis* was grown at 30 °C in GM17 medium (BD, Difco) supplemented with glucose, using either liquid or solid formulations. Erythromycin (5 µg/mL) was added when required. *Escherichia coli* strains were cultured aerobically at 37 °C in LB medium. When appropriate, ampicillin (100 µg/mL), kanamycin (25 µg/mL), or erythromycin (200 µg/mL) was included. *Salmonella enterica* serovar Typhimurium 92–35 was cultured in BHI broth (Difco, BD) at 37 °C with shaking. *Listeria monocytogenes* EGD was grown statically in BHI broth at 25 °C.

**Table 1.** Bacterialstrains and plasmids used in this study.

Strain or plasmid	Description	Reference
**Strain**		
*L. ruminis* ATCC 25644 (WT)	Isolated from human intestine	ATCC
*L. ruminis* BKN502 (Mot)	A motility-acquired mutant derived from ATCC 25644	This study
*L. lactis* NZ9000	Donor of conjugation with pGMβ1	[61]
*L. acidophilus* NCFM	Isolated from human intestine	[62]
*L. reuteri* DSM 20016^T^	Isolated from human intestine	DSMZ
*L. agilis* BKN88	Isolated from avian gut	[15]
*S*. Typhimurium 92–35	Isolated from human intestine	[15]
*L. monocytogenes* EGD	Isolated from beer malt and beer wort	[63]
*E. coli* SG13009	Cloning host for pQE30 and pRep4	Qiagen
Clear coli® BL21	Cloning host, LPS free	Lucigen Co.
*E. coli*HST08	Cloning host for pGMβ1	Takara
**Plasmid**		
pQE30	Amp^r^, His-tagged protein expression vector	Qiagen
pREP4	Km^r^, *lacI*	Qiagen
pGMβ1	Em^r^, Amp^r^, Conjugal plasmid	[59]
pTRK882	PCR template of *pgm* promoter	[58]

### Isolation of a motility-acquired mutant

*Ligilactobacillus ruminis*ATCC 25644 was obtained from the American Type Culture Collection (ATCC), and isolation of a motility-acquired mutant was attempted. A 1.5 mL volume of MRS soft agar medium containing 0.15% agar was dispensed into 24-well plates. The center of each well was inoculated by stab-inoculation with a culture of *L. ruminis* ATCC 25644. The plates were then incubated anaerobically at 37 °C for 24 hours. After incubation, a small amount of soft agar was collected from regions distant from the stab site and streaked onto fresh MRS agar plates. Individual colonies that formed were then stab-inoculated into fresh soft agar medium to assess motility. Colonies that exhibited clear motility were selected and purified for further analysis.

### Growth, motility, and flagella observation

The growth of *L. ruminis* was evaluated by measuring optical density at 600 nm (OD₆₀₀) and by colony-forming unit (CFU) enumeration. Motility was assessed by inoculating colonies onto MRS soft agar plates and incubating overnight to observe bacterial spreading and growth. For liquid cultures, motility was also examined directly by light microscopy. Flagellar filaments were visualized by staining with FLAGELLA STAIN (Hardy Diagnostics) and observed under a BZ-X710 optical microscope (Keyence). Transmission electron microscopy (TEM) of negatively stained flagella, using ammonium molybdate, was performed by Hanaichi Ultrastructure Research Institute (Aichi, Japan) using a JEOL JEM-1400Flash microscope operated at 100 kV.

### Chemotaxis assay

In previous work with chemotactic and non-chemotactic (Δ*cheA*) *Ligilactobacillus agilis* strains, the microscopic agar-drop assay proved more reproducible than the capillary assay for assessing chemotaxis.^13^ Accordingly, chemotaxis in *L. ruminis* was assessed using the agar-drop assay as described by Suzuki et al.^13^. Test conditions included variations in pH and the presence of bile salts. A drop of agar containing the test compound was placed onto a microscope slide and covered with a cover glass. The surrounding area was then filled with a suspension of bacterial cells. Images near the agar drop were captured for 10 minutes using a BZ-X710 optical microscope (Keyence), and the number of cells was quantified using ImageJ software. Additionally, bacterial suspensions were mixed directly with test solutions, and cell behavior was tracked for 20 seconds using the motion analysis software VW-H2MA.

### Acid and bile salt tolerance assays

Acid tolerance was assessed following the method described by Liong et al.^40^. Late-log-phase bacterial cultures were inoculated at 5% (v/v) into MRS medium adjusted to pH 3.0. At 30-minute intervals, aliquots of the culture were collected, and viable cell counts were determined by plating on MRS agar. Bile tolerance was evaluated according to the method of Pfeiler et al.^41^. Late-log-phase cultures were serially diluted and plated onto MRS agar containing sodium deoxycholate at final concentrations of 0%, 0.25%, or 0.5%. Colony formation was examined to assess growth under each condition. As a reference strain, *Lactobacillus acidophilus* NCFM—reported to be tolerant to both acid and bile salts—was included in both assays.^42^

### Preparation of recombinant proteins

The major flagellins of *L. ruminis* ATCC 25644 are encoded by *fliC1* (NQ504_08700) and *fliC2* (NQ504_08730), which produce proteins with identical amino acid sequences. Therefore, they are collectively referred to as FliC1/2. Total genomic DNA was crudely extracted from bacterial cells and used as the template for PCR amplification of the *fliC1/2* fragment with primers DOKJ1993 and DOKJ1994. PCR was performed using KOD One® PCR Master Mix (TOYOBO). The PCR product and pQE30 vector (QIAGEN) were digested with *Kpn*I and *Hind*III (Takara), ligated, and transformed into *E. coli* SG13009 (pREP4). Positive clones were confirmed by colony PCR using EmeraldAmp PCR Master Mix (TaKaRa) with primers DOKJ215 and DOKJ216, followed by Sanger sequencing. Recombinant His-tagged proteins were expressed and purified from *E. coli* following the manufacturer’s protocol provided by QIAGEN. To eliminate the influence of lipopolysaccharide (LPS) in cell-based assays, plasmids were also introduced into ClearColi BL21, and recombinant proteins were purified from this strain. Protein production and purification were confirmed by SDS-PAGE and Coomassie Brilliant Blue (CBB) staining. In addition to *fliC1/2*, another flagellin gene, *fliC3* (NQ504_03640), located distally from the motility gene cluster in the *L. ruminis* ATCC 25644 genome, was also cloned. Recombinant full-length FliC3 (His-FliC3) and a FliC3-specific variable region fragment (His-F3VR) were prepared. PCR amplification was performed using genomic DNA and primers DOKJ1991/DOKJ1992 or DOKJ2029/DOKJ2030, respectively. Recombinant proteins were prepared using the same strategy as for FliC1/2. Notably, FliC3 could not be solubilized in 8 M urea; therefore, purification was performed using CelLytic™ B Cell Lysis Reagent. A solubilization buffer containing 0.5% SDS, 8 M urea, 100 mM NaH₂PO₄, and 10 mM Tris-Cl (pH 8.0) was used to improve protein solubility. The recombinant major pilin protein LrpA, reported to be a primary component of pili in *L. ruminis* ATCC 25644,^5^ was also prepared. The *lrpA* gene fragment was amplified from total DNA using primers DOKJ2083 and DOKJ2084 and purified using the same procedure as for FliC1/2 to obtain His-LrpA. The primer sequences are listed in Table S1.

### Amino acid sequence comparison and structural prediction of flagellin proteins

A sequence alignment of FliC1/2 and FliC3 amino acid sequences was performed using Clustal Omega. To predict the three-dimensional structures of the proteins, each sequence was submitted to AlphaFold3.

### Preparation of antibodies

Purified recombinant proteins—His-FliC1/2, His-FliC3, His-F3VR, and His-LrpA—were adjusted to a concentration of 100 μg/mL and mixed at a 1:1 ratio with Freund’s Complete Adjuvant to prepare water-in-oil emulsions. The emulsions were administered intraperitoneally to BALB/c or C3H/HeN mice (CLEA Japan). Two booster injections were given at two-week intervals using Freund’s Incomplete Adjuvant. Serum samples were collected following the final immunization. All animal experiments were approved by the Animal Care and Use Committee of Tokyo University of Agriculture.

### Detection of flagellin and pilus proteins

For flagellin detection, bacterial cells were collected from overnight cultures of *L. ruminis* and resuspended in 8 M urea solution. After centrifugation, the supernatant was mixed with an equal volume of 2 × Laemmli buffer. Samples were subjected to SDS-PAGE followed by Coomassie Brilliant Blue (CBB) staining or transferred to a PVDF membrane for western blotting. Flagellin was detected using anti-FliC1/2 or anti-FliC3 sera, followed by HRP-conjugated anti-mouse IgG antibodies (Sigma). For detection of pilus proteins, *L. ruminis* cells grown on agar plates were collected and suspended in PBS. After centrifugation, cells were resuspended in 8 M urea solution, and disrupted using glass beads with a FastPrep instrument (MP Biomedicals). The resulting supernatant was collected by centrifugation, and protein concentration was determined. Samples were then mixed with 2 × Laemmli buffer and analyzed by SDS-PAGE, CBB staining, and western blot using LrpA-specific antisera.

### Isolation of flagella

Flagella were isolated from *L. ruminis*, *L. agilis*, *Salmonella* Typhimurium, and *Listeria monocytogenes* according to previously described methods.^15^^,^^43-47^ Bacterial cells from logarithmic phase or overnight cultures were harvested and resuspended in sterile distilled water, followed by mechanical shearing for 1 min using Crush Milcer (Iwatani). The suspensions were centrifuged at 8,000 × g for 5 minutes to remove cells, and the supernatants were then subjected to ultracentrifugation at 100,000 × g for 60 minutes. The resulting pellets were resuspended in sterile distilled water and stored at 4 °C or −20 °C until use.

### Immunofluorescence microscopy

Immunofluorescence staining was performed following previously described protocols.^48,49^ Bacterial cells grown on agar plates were suspended in PBS and incubated in a 1:100 dilution of anti-LrpA antiserum. After washing, the cells were labeled with a 1:200 dilution of Alexa Fluor™ 594-conjugated goat anti-mouse IgG (Thermo Fisher Scientific). Stained samples were observed using a BZ-X710 optical microscope (Keyence).

### Immunogold electron microscopy

Immunogold labeling was performed based on previously described methods.^50,51^ Bacterial cells grown on agar or in liquid culture were harvested and suspended in PBS, then applied onto hydrophilic carbon-coated copper grids (STEM). After blocking with 1% BSA in PBST, the grids were incubated with a 1:50 dilution of anti-LrpA or anti-FliC3 antiserum prepared in the same buffer. Following washing steps, the grids were incubated with a 1:10 dilution of Immunogold conjugate anti-mouse IgG (H + L)-10 nm (BBI Solutions). Finally, the samples were negatively stained with 2% ammonium molybdate and observed using a transmission electron microscope (H−7600, Hitachi).

### Assessment of proinflammatory activity using cultured cells

Proinflammatory responses were assessed based on previously described methods,^24,45^ including quantification of IL−8 production by Caco−2 cells and a reporter gene assay using HEK-Blue hTLR5 cells (InvivoGen). Caco−2 cells were cultured in DMEM (Wako) supplemented with 10% fetal bovine serum (FBS), non-essential amino acids, GlutaMAX (Thermo Fisher Scientific), and penicillin/streptomycin. Cells were maintained at 37 °C in a 5% CO₂ incubator. Cells were seeded at 2.0 × 10⁴ cells/well in 96-well plates and incubated overnight. Washed *L. ruminis* cells (1.0 × 10⁴–10⁶ CFU/well) or purified flagellins (0.1–100 nM) were added to the wells and incubated for 12 hours. IL−8 concentrations in the culture supernatants were quantified using the OptEIA™ Human IL−8 ELIZA Set (BD). HEK-Blue hTLR5 cells were cultured according to the manufacturer's instructions. Cells were seeded at 1.2 × 10⁵ cells/well and stimulated with *L. ruminis* cells (1.0 × 10⁴–10⁶ CFU/well) or flagellin (0.01–1 nM) for 16 hours. SEAP activity was measured by assessing absorbance at 650 nm using a microplate reader (CORONA ELECTRIC).

### Adhesion assay to fibronectin and mouse intestinal mucus

Fibronectin Neosilk, Cellular (IBL) was purchased for use in the assay. Mouse intestinal mucus was prepared according to previously described methods.^52,53^ The small intestine was excised from euthanized 9-week-old female BALB/c mice (CLEA Japan), opened longitudinally, and washed with PBS to remove luminal contents. Mucus was then gently scraped from the mucosal surface using the edge of a microscope slide and transferred to a 50 mL tube. The mucus was suspended in 10 mL of 7 M guanidine hydrochloride (Gu-HCl) and incubated at room temperature for 5 days. After incubation, the sample was centrifuged at 20,000 × g for 60 minutes, and the supernatant was collected. Adhesion assays were conducted based on previously reported methods with minor modifications.^4,20^ Fibronectin and mucus extracts were diluted in PBS to final concentrations of 0.1 mg/mL and 0.5 mg/mL, respectively, and 200 μL of each was added to wells of a 96-well cell culture-treated flat-bottom microplate. Plates were incubated overnight at 4 °C for coating. After blocking with 2% BSA, *L. ruminis* cultures (OD₆₀₀ = 1.0) concentrated 3-fold were added at 100 μL per well and incubated for 5 hours under anaerobic conditions. After washing with PBS, adherent bacteria were stained with crystal violet, and absorbance was measured to quantify adhesion. As a positive control, *Limosilactobacillus reuteri* DSM 20016 (formerly *Lactobacillus reuteri*), a strain known for adhesion to intestinal epithelial cells, was used.^54^

### Antibiotic-assisted colonization of *L. ruminis* in mice

Exogenous bacterial colonization is generally inefficient in animals with intact gut microbiota, and colonization of a non-native host species is particularly challenging. While germ-free mice provide a model system, their immature immune system limits interpretation. Therefore, we adopted an antibiotic-assisted colonization model based on previously reported methods.^55-57^ A 100 μL aliquot of *L. ruminis* culture was spread on MRS agar supplemented with 100 μg/mL streptomycin and 25 μg/mL kanamycin. Colonies that grew under these conditions were isolated for subsequent experiments. Seven-week-old female BALB/c mice (CLEA Japan) were provided with sterilized drinking water containing an antibiotic cocktail (0.5 g/L ampicillin, 1 g/L vancomycin, 1 g/L neomycin) for 10 days. Feces were collected every 3–4 days, and total gut bacterial load was monitored by anaerobic culture on GAM agar. In most mice, bacterial counts fell below the detection limit. The antibiotic-treated water was then replaced with sterilized water containing 0.5 g/L streptomycin and 0.25 g/L kanamycin for an additional 2 days. On day 13, 1.0 × 10⁹ CFU of the antibiotic-resistant *L. ruminis* strain was administered intragastrically. Fecal bacterial counts and body weight were monitored every 3–4 days. Total bacterial counts were assessed by plating on GAM agar, while lactobacilli were quantified using MRS agar supplemented with streptomycin and kanamycin. All culturing was performed anaerobically. Colonies formed on MRS agar were randomly isolated and identified using MALDI-TOF MS (BD™ Bruker MALDI Biotyper™ sirius). Fecal samples were also subjected to DNA extraction after bead-beating. PCR amplification of *L. ruminis*-specific DNA fragments was performed using MightyAmp DNA Polymerase Ver.3 (Takara) and primers DOKJ1993 and DOKJ1994 targeting *fliC1/2*. 16S rRNA was used as a reference gene (primers DOKJ200 and DOKJ201). One month after bacterial administration, mice were euthanized and serum, cecal contents, and intestinal tissues were collected. Serum IgG titers specific to *L. ruminis* lysates were quantified by ELIZA using HRP-conjugated anti-mouse IgG antibodies. Plasma TNF-*α* levels were measured using the OptEIA™ Mouse TNF ELIZA Set (BD). Intestinal length was also measured. Cecal contents were snap-frozen in liquid nitrogen immediately after dissection and stored for later analysis. Mice were divided into three groups (saline control, WT, and Mot), with five animals per group. Antibiotic-containing drinking water was replaced every three days. The experiment was repeated independently twice to confirm reproducibility.

### Genome sequencing of the motility-acquired mutant

The motility-acquired mutant of *L. ruminis* ATCC 25644 was cultured in MRS broth to the logarithmic phase, harvested by centrifugation, and washed. Cells were resuspended in Tris-HCl buffer containing 10 mg/mL lysozyme and 20 U/mL mutanolysin and incubated at 37 °C for 30 minutes. After pelleting, cells were resuspended in 20 mM Tris-HCl containing 5 mM EDTA, 1% SDS, and 100 μg/mL Proteinase K, followed by incubation at 65 °C for 1 hour. Proteins were removed by phenol-chloroform extraction, and nucleic acids were precipitated with 0.3 M sodium acetate and isopropanol. The DNA pellet was washed with 70% ethanol and dissolved in TE buffer. After RNase treatment, pure genomic DNA was obtained by ethanol precipitation. Whole-genome sequencing and mutation site identification were performed by GenomeRead Co., Ltd. (Takamatsu, Japan) using the NovaSeq 6000 platform. Library preparation was conducted using the Illumina DNA Prep Tagmentation Kit according to the manufacturer's instructions. Sequencing reads were quality-filtered, and mutations were identified using Breseq with the complete genome of *L. ruminis* ATCC 25644 (GenBank: CP102284.1) as the reference. Sequence coverage of the sequencing was 740-fold.

### RNA-Seq analysis

*L. ruminis* ATCC 25644 and its motility-acquired mutant were cultured in 5 mL of MRS broth to the logarithmic growth phase, at which motility was evident. Cells were harvested by centrifugation, and total RNA was extracted using the RNeasy Mini Kit (QIAGEN). RNA sequencing and subsequent analysis were outsourced to Macrogen Japan (Tokyo, Japan). Sequencing was performed on the Illumina NovaSeq 6000 platform. Library preparation was conducted using the Illumina Stranded Total RNA Prep Kit along with the Ribo-Zero Plus rRNA Depletion Kit, following the manufacturer’s instructions. Sequencing reads were aligned to the *L. ruminis* reference genome (assembly ASM2515089v1; GenBank accession: GCA_025150895.1) using the Bowtie2 aligner. Transcripts and gene models were assembled with StringTie based on the reference genome annotation. Gene and transcript expression levels were quantified as raw read counts, and normalized expression was calculated as FPKM (Fragments Per Kilobase of transcript per Million mapped reads) and TPM (Transcripts Per Kilobase Million). For differential expression analysis, the raw read count data obtained using the -e option of StringTie were used as input (2,144 genes). During preprocessing, low-quality transcripts were filtered, and data were normalized using the TMM (trimmed mean of M values) method. A total of 2,108 genes remained after filtering. Statistical analysis was performed using the edgeR package. Differentially expressed genes (DEGs) were identified based on a threshold of absolute fold change ≥2 and an exactTest raw *p*-value <0.05. This analysis resulted in 461 significant DEGs.

### Cloning of conjugative expression plasmids and genetic transformation of *L. ruminis*

A constitutive promoter derived from the phosphoglycerate mutase gene of *Lactobacillus acidophilus* NCFM (*Ppgm*) was used as a constitutive expression element.^58^ The *Ppgm* region was amplified from pTRK882 using Primer 1 and Primer 2, as listed in Table S2. For amplification of putative transcriptional regulator genes, total DNA extracted from wild-type *L. ruminis* ATCC 25644 was used as a template, and Primer 3 and Primer 4 were used. After purification, the *Ppgm* region and the transcriptional regulator gene fragment were fused by overlap-extension PCR using Primer 1 and Primer 4. All PCR reactions were performed using KOD One® PCR Master Mix (TOYOBO). The conjugative plasmid pGMβ1 (59) was linearized by *Apa*I digestion (TaKaRa), and circular plasmids were constructed using the In-Fusion® HD Cloning Kit (TaKaRa) with the fused PCR products. The resulting constructs were transformed into *E. coli* HST08 Premium Competent Cells. Colony PCR was performed using EmeraldAmp PCR Master Mix (TaKaRa) and primers DOKJ1964 and DOKJ1965 to screen for clones carrying the expected transcriptional regulator expression cassette inserted into pGMβ1. Plasmids were extracted from positive clones using the NucleoSpin Plasmid EasyPure kit (Macherey-Nagel) and verified by Sanger sequencing. To confirm the integrity of the entire plasmid, PCR was conducted using nine primer sets (DOKJ1398 to DOKJ1415). Validated plasmids were introduced into *Lactococcus lactis* NZ9000 by electroporation. Successful transformation of *L. lactis* was confirmed by colony PCR using EmeraldAmp PCR Master Mix (TaKaRa) with primers DOKJ1964 and DOKJ1965. Conjugative transfer of plasmids from *L. lactis* to *L. ruminis* was performed following previously described methods,^59,60^ using either a plate-mating method—in which early-log phase cultures were co-incubated on MRS agar—or a filter-mating method using MF-Millipore™ 0.45 μm MCE membranes (Sigma). PCR with primers DOKJ1964 and DOKJ1965 was used to confirm successful plasmid transfer to *L. ruminis*. Total RNA was extracted from plasmid-harboring *L. ruminis* using the NucleoSpin RNA kit (Macherey-Nagel), and expression of the target gene was confirmed by RT-PCR using PrimeScript™ RT Reagent Kit (TaKaRa) with Primer 3 and Primer 4. To ensure the absence of genomic DNA contamination, PCR was also performed using TaKaRa Ex Taq (TaKaRa) with the same primer set.

## Results

### Isolation and characterization of a motility-acquired mutant strain

**Figure 1.**
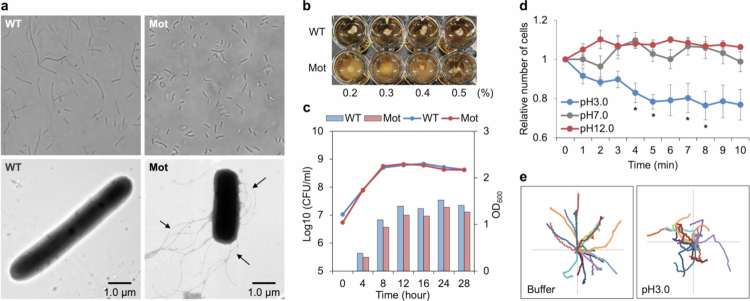
Phenotypic characterization of a motility-acquired mutant derived from *L. ruminis* ATCC 25644. a, Cell morphology of the wild-type (WT, left) and motility-acquired mutant (Mot, right). Top: phase-contrast microscopy ( × 1,000); bottom: transmission electron microscopy (TEM) with negative staining ( × 6,000; scale bar: 1 μm). Arrows indicate flagella. b, Motility assay on soft agar (MRS medium supplemented with 0.2−0.5% agar). c, Bacterial growth curve based on CFU enumeration (line graphs) and turbidity measurement (bar graphs). Blue and red indicate WT and Mot, respectively. *n* = 3; data are shown as mean ± standard error (SE). d, Chemotaxis assay (agar drop assay) of the Mot strain. Relative cell densities near agar drops of pH 3.0 (blue), pH 7.0 (gray), and pH 12.0 (red). *n* = 3; mean ± SE; Significant differences between the pH 7.0 and other pH values are indicated with an asterisk (*P < 0.05; Dunnett’s test). e, Tracking of individual Mot cells in neutral (buffer) and acidic (pH 3) solutions. Each cell trajectory is plotted with (x, y) = (0, 0) as the starting point. *n* = 20.

A screening using MRS soft agar medium led to the isolation of a spontaneous motility-acquired mutant (BNK502; Mot) derived from *Lactobacillus ruminis* ATCC 25644 (wild type; WT). The Mot strain formed flagella and exhibited clear motility during the logarithmic growth phase (Figure 1ab; Movie S1, 2). Compared to the WT, Mot cells tended to be shorter in length (Figure 1a). Although both strains showed similar growth curves, the optical density of Mot cultures was consistently lower (Figure 1c). In chemotaxis assays, the Mot strain exhibited an increased frequency of tumbling in acidic solution (pH 3), and the number of cells surrounding the acidic agar drop decreased over time (Figure 1d, e). This decrease indicates that the cells actively moved away from the acid. These findings suggest that the Mot strain exhibits negative chemotaxis against acidic conditions.

### Flagellin production and proinflammatory activity

**Figure 2.**
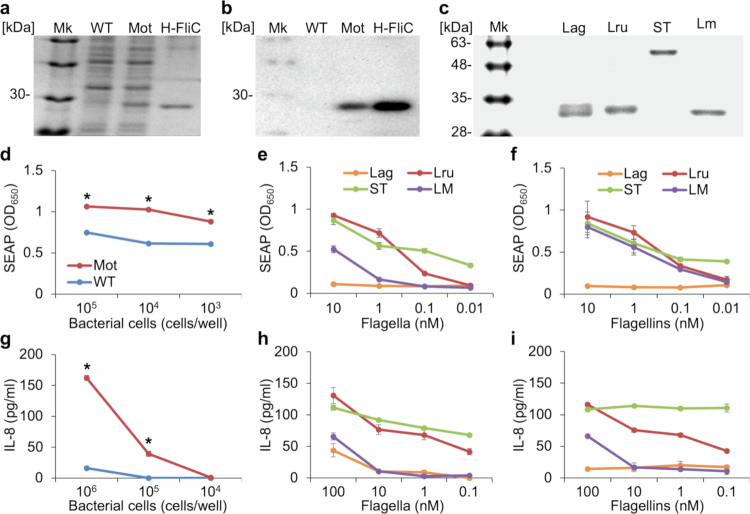
Flagellin expression and proinflammatory activity of *L. ruminis* Mot strain. a, Profiling of surface-associated proteins extracted from *L. ruminis*. Mk, protein size marker; H-FliC, purified His-tagged FliC1/2. b, Western blot analysis using anti-FliC1/2 antibodies. **c**, Purified flagellins from various bacterial species: Lag, *L. agilis*; Lru, *L. ruminis* Mot strain; ST, *Salmonella Typhimurium*; LM, *Listeria monocytogenes*. d–f, Reporter gene assays using HEK-Blue hTLR5 cells stimulated with whole cells (d), intact purified flagella (e), or monomerized purified flagellin (f). The secreted alkaline phosphatase (SEAP) was measured. g–i, IL−8 production by Caco−2 cells stimulated with whole cells (g), intact purified flagella (h), or monomerized purified flagellin (i). IL−8 levels in the culture supernatants were quantified. *n* = 3; data are shown as mean ± standard error (SE). d, g, Significant difference between WT and Mot strains is indicated using an asterisk (*P < 0.0001; Two-way ANOVA with Bonferroni multiple comparisons test.).

Cell surface proteins were extracted from both the WT and Mot strains, and flagellin bands were visualized by SDS-PAGE followed by Coomassie Brilliant Blue (CBB) staining. In the Mot extract, a distinct band was observed at approximately 30 kDa, corresponding to the molecular weight of His-tagged FliC1/2 (Figure 2a). Since the theoretical molecular weight of FliC1/2, based on its amino acid sequence, is approximately 29 kDa and no corresponding band was detected in the non-flagellated WT strain, this band was identified as the FliC1/2 protein. Western blot analysis also confirmed the presence of a specific band at the same position as His-FliC1/2 only in the Mot extract, further supporting flagellin production in the Mot strain (Figure 2b).

Although FliC3 shares sequence similarity with FliC1/2 at the *N*- and C-terminal regions, it contains unique sequences in the central region (Fig. S1ab). While SDS-PAGE and CBB staining did not clearly detect a band corresponding to FliC3, a FliC3-specific band was observed by western blotting in the Mot strain (Fig. S1c). Additionally, immunoelectron microscopy revealed signals suggestive of FliC3 localization within the flagellar filaments (Fig. S1d). These results indicate that FliC3 is produced in the Mot strain but in significantly smaller amounts compared to FliC1/2, suggesting that it is a minor flagellin.

Reporter assays using HEK-Blue hTLR5 cells revealed strong activation in response to the Mot strain, whereas the WT strain showed minimal activity (Figure 2d). Similarly, stimulation of Caco−2 cells with the Mot strain induced IL−8 production in a cell number-dependent manner (Figure 2g). Tests using isolated flagellar filaments and monomerized flagellins from Mot also demonstrated dose-dependent responses mediated via TLR5 (Figure 2cefhi). For comparison, flagellar filaments and flagellins derived from *L. agilis*, *Salmonella* Typhimurium, and *Listeria monocytogenes* were also examined. The *L. ruminis*-derived flagellins exhibited remarkably higher proinflammatory activity than those from the closely related *L. agilis*, and were comparable to or greater than those from *L. monocytogenes*. Additionally, flagellins prepared from pig-derived *L. ruminis* KJCC103 and horse-derived *L. ruminis* BKN207 strains stored in our laboratory showed similar levels of activity, regardless of the host origin (Fig. S2). In contrast, FliC3, the minor flagellin, did not exhibit any detectable proinflammatory activity (Fig. S1e).

### Pilus formation and adhesion properties of *L. ruminis* strains

**Figure 3.**
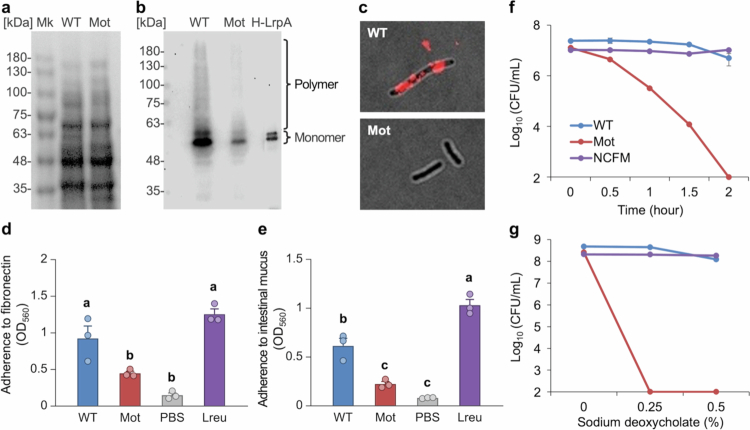
Pilus expression, adhesion, and acid/bile tolerance of *L. ruminis.* a, Total protein profiling of bacterial lysates. Mk, protein size marker. b, Detection of pili by western blot using LrpA-specific antibodies. H-LrpA, purified His-tagged LrpA protein. c, Immunofluorescence staining using LrpA-specific antibodies. Magnification: × 1000, excitation wavelengths:470/40, emission wavelengths:525/50. d, e, Adhesion assays to fibronectin (d) and mouse intestinal mucus. Lreu, *L. reuteri*. (e). *n* = 3, mean ± SE; One-way ANOVA with Tukey multiple comparisons test; no significant differences between groups among the same letter (*P* ≥ 0.05). f, g, Acid tolerance (f) and bile salt tolerance (g) assays. *n* = 3, mean ± SE.

Total cellular proteins were extracted from both the WT and Mot strains, and pilus expression was analyzed by western blotting (Figure 3ab). In the Mot strain, both the ladder-like bands derived from polymerized pilin subunits and the monomeric band of the major pilin, LrpA, were markedly reduced. Consistently, LrpA-specific immunofluorescence staining revealed clear fluorescence signals on the cell surface of WT cells, whereas no such signals were detected in the Mot strain (Figure 3c). Electron microscopy was also performed on WT cells labeled with LrpA-specific antibodies and gold colloid; although the images were not very clear, filamentous structures were observed (Fig. S3). These results indicate a significant reduction in pilin protein expression and pilus formation in the Mot strain.

Adhesion assays using purified fibronectin and mucosal extracts prepared from mouse intestines demonstrated that the Mot strain exhibited significantly lower adhesion capacity compared to the WT (Figure 3de). These findings suggest a correlation between pilus formation and adhesion in *L. ruminis*, whereas flagellum formation and motility are unlikely to play a significant role in adhesion.

### Acid and bile acid tolerance

We assessed acid and bile tolerance in *L. ruminis* to model intestinal stresses, motivated by our prior observation that motile *L. agilis* are less tolerant to acid and bile than non-motile lactobacilli.^13^ In acid tolerance assays, the WT strain maintained a high viable cell count for at least 2 hours. In contrast, the Mot strain exhibited a time-dependent decrease in viability and was completely nonviable after 2 hours of exposure (Figure 3f). In bile acid tolerance tests, the WT strain was able to grow in MRS medium containing 0.5% sodium deoxycholate, whereas the Mot strain failed to grow in media containing 0.25% or higher concentrations of sodium deoxycholate (Figure 3g). These findings indicate that the Mot strain exhibits significantly reduced tolerance to both acidic conditions and bile acids compared to the WT strain.

### Intestinal colonization of *L. ruminis* strains in mice

**Figure 4.**
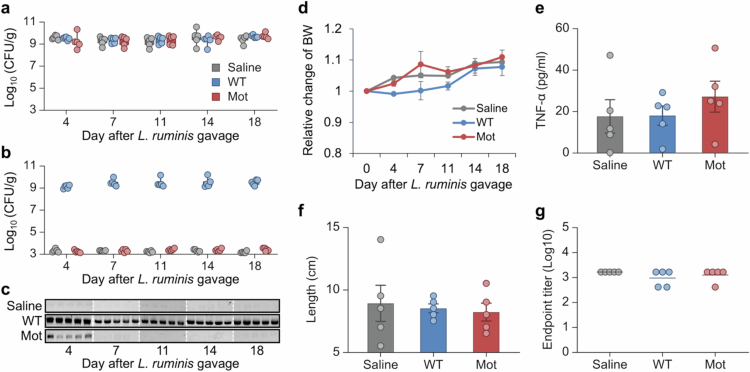
Colonization of *L. ruminis* in the mouse intestine and evaluation of inflammation-related parameters. a, b, Enumeration of viable fecal bacteria over time. Anaerobic culture on GAM agar (a) and MRS agar (b). Each symbol represents an individual mouse (5 mice/group). c, Detection of *L. ruminis* in fecal samples by species-specific PCR. DNA was extracted from feces collected for viable count assays. d, Time-course of relative body weight changes. Mean ± SE. e, Quantification of TNF-*α* in serum. Mean ± SE. f, Comparison of intestinal lengths. Mean ± SE. g, Measurement of *L. ruminis*-specific antibody titers. No significant differences detected by Tukey’s multiple comparison test (d–g).

Mice pre-treated with an antibiotic cocktail to deplete the gut microbiota were orally administered either of the *L. ruminis* strains. Non-selective culturing on GAM medium revealed no significant differences in total viable bacterial counts among groups, suggesting that a comparable level of gut microbiota was present across all mice (Figure 4a). Identification of colonies formed on GAM medium showed the presence of *Citrobacter amalonaticus* in all groups. In contrast, selective culturing on MRS medium showed that the WT strain persisted in fecal samples, with colony counts exceeding 10⁹ CFU maintained for 18 days. However, colonies from the Mot group were no longer detectable within four days post-administration (Figure 4b). Random identification of colonies from the WT group confirmed all isolates as *L. ruminis*. Furthermore, *L. ruminis*-specific genes were consistently detected by PCR from DNA extracted directly from fecal samples throughout the experimental period (Figure 4c). These results indicate that only the WT strain was capable of stable colonization in the mouse intestine, whereas the Mot strain lacked colonization ability. To assess whether colonizing WT cells produced flagellin in vivo, we measured flagellin activity in fecal extracts using HEK-Blue hTLR5 reporter cells. Although limited by the availability of fecal samples from only a subset of animals, no significant differences in activity were observed compared to the saline control group, providing no evidence for additional flagellin production by WT in vivo (Fig. S4). To evaluate potential inflammation triggered by flagellin, body weight gain, plasma TNF-*α* levels, intestinal length, and *L. ruminis*-specific antibody titers were measured. None of these parameters showed significant differences among groups (Figure 4d–g).

To confirm reproducibility, the experiment was repeated twice with consistent results (Fig. S5). A notable exception was observed in the other trial, where colonies appeared on MRS agar plates from the Mot group after day 5 and persisted throughout the experiment. However, random identification revealed that these colonies were *Ligilactobacillus murinus*, a known murine commensal. It is likely that drug-resistant clones of this species emerged during the experiment. Consistently, PCR analysis failed to detect *L. ruminis*, reinforcing the conclusion that the Mot strain is unable to colonize the mouse intestine.

### Genome sequencing and transcriptomic analysis of the motility-acquired mutant

**Figure 5.**
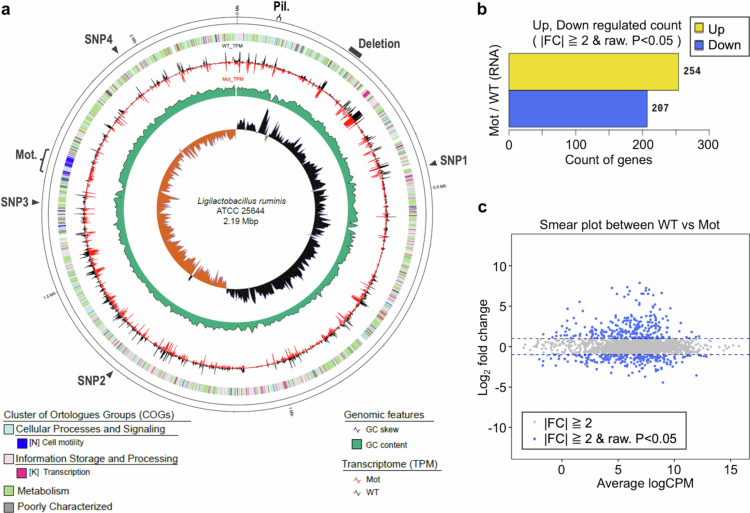
Genome sequencing and transcriptomic analysis of the Mot strain. a, Genome map of *L. ruminis* ATCC 25644 showing mutation sites. Deletion regions, point mutations (SNP1–4), the pilus operon (Pil.), and motility gene cluster (Mot.) are indicated. Details of point mutations and genes within the deletion region are listed in Table 1 and Table 2, respectively. b, Numbers of genes significantly upregulated or downregulated in Mot. **c**, Smear plot comparing gene expression levels; upregulated genes are plotted above the baseline and downregulated genes below.

Genome sequencing of the Mot strain revealed a ~35 kb deletion and four point mutations (Figure 5a). As listed in Table 2, the point mutations were located within sequences annotated as a transposase, an RNA-binding protein, a non-coding region, and GMP synthase (*guaA*). The mutation in *guaA* was a nonsense mutation, suggesting potential disruption of gene function; however, it is unlikely to be directly associated with motility. Therefore, these point mutations are not considered to be the primary cause of motility acquisition in the Mot strain. In contrast, the 35 kb deletion encompassed 34 coding sequences, and it was deemed likely that the loss of one or more genes within this region contributed to the acquisition of motility (Table 3). Notably, this deleted region included five predicted transcriptional regulators: RRTF, DegV, GntR1, ROK, and GntR2.

**Table 2.** Point mutations of genome sequencing.

ID	Locus	Annotation	Mutation
SNP1	NQ504_02085	IS200/IS605 family transposase, tnpA	CTG→ATG L143M
SNP2	NQ504_06730	S1‑like domain‑containing RNA‑binding protein	CGG→CGT R119R
SNP3	(NQ504_08510) (NQ504_08515)	Intergenic region between IS256 family transposase and alpha‑amylase family glycosyl hydrolase	C→A
SNP4	NQ504_09810	Glutamine‑hydrolyzing GMP synthase, guaA	TAC→TAA Y105*

**Table 3.** Genes included in the 35 kb-deletion.

Locus	Annotation (Abbreviation in this study)
NQ504_01000	PTS sugar transporter subunit IIC
NQ504_01005	alpha/beta hydrolase
NQ504_01010	glutamate 5-kinase
NQ504_01015	glutamate−5-semialdehyde dehydrogenase
NQ504_01020	NADPH-dependent oxidoreductase
NQ504_01025	glycosyltransferase family 8 protein
NQ504_01030	SAM-dependent methyltransferase
NQ504_01035	response regulator transcription factor (RRTF)
NQ504_01040	ATP-binding protein (ATPbp)
NQ504_01045	D-alanyl-D-alanine carboxypeptidase
NQ504_01050	YhgE/Pip domain-containing protein
NQ504_01055	DegV family protein (DegV)
NQ504_01060	DUF1836 domain-containing protein
NQ504_01065	hypothetical protein
NQ504_01070	TrkH family potassium uptake protein
NQ504_01075	AzlC family ABC transporter permease
NQ504_01080	AzlD domain-containing protein
NQ504_01085	alpha/beta hydrolase
NQ504_01090	hypothetical protein
NQ504_01095	IS256 family transposase
NQ504_01100	GntR family transcriptional regulator (GntR1)
NQ504_01105	hypothetical protein
NQ504_01110	acetylxylan esterase
NQ504_01115	glycoside hydrolase family 1 protein
NQ504_01120	PTS sugar transporter subunit IIC
NQ504_01125	PTS sugar transporter subunit IIB
NQ504_01130	PTS lactose/cellobiose transporter subunit IIA
NQ504_01135	transposase
NQ504_01140	ROK family protein (ROK)
NQ504_01145	glycoside hydrolase family 1 protein
NQ504_01150	biotin/lipoyl-binding protein
NQ504_01155	transposase
NQ504_01160	ATP-binding cassette domain-containing protein
NQ504_01165	GntR family transcriptional regulator (GntR2)
NQ504_01170	ATP-binding cassette domain-containing protein

To comprehensively investigate gene expression changes between the WT and Mot strains, transcriptomic analysis was performed using RNA sequencing. A total of 254 genes were significantly upregulated and 207 genes were significantly downregulated in Mot compared to WT (Figure 5bc). Among the most strongly upregulated genes, a majority were associated with motility and chemotaxis (Supporting Information, S1). In contrast, transcriptional regulators known to suppress motility (NQ504_02240 and NQ504_08745) were among the most downregulated genes. The list of highly downregulated genes also included those involved in carbohydrate and glycogen metabolism (*glgA*, *glgB*, *glgD*, *α*-amylase family glycosyl hydrolases, etc.), biotin metabolism (bioY, biotin--[acetyl-CoA-carboxylase] ligase), and ABC transporters. Reduced expression of a choloylglycine hydrolase family protein might contribute to the increased bile sensitivity observed in the Mot strain. While expression levels of pilus-related genes were not drastically altered, they were consistently lower in the Mot strain. It is possible that more pronounced differences may be observed during stationary phase or under solid culture conditions. Importantly, the majority of genes within the deleted region in Mot were actively expressed in the WT strain, including all five of the aforementioned predicted transcriptional regulators.

### Suppression of motility and flagellum formation by complementation of deleted genes

**Figure 6.**
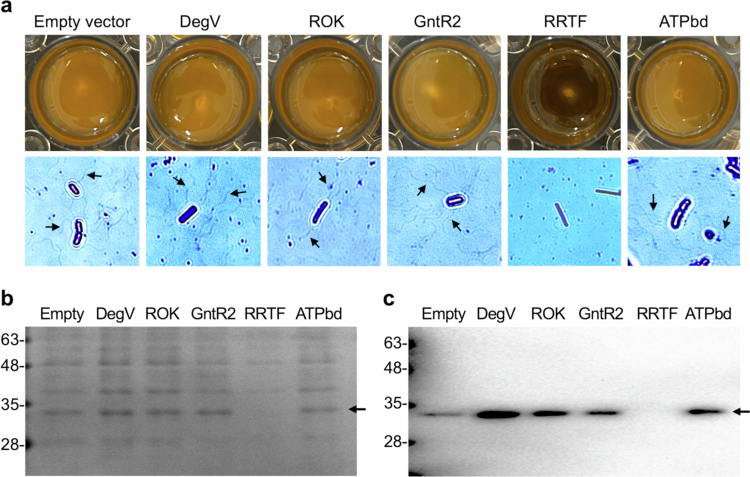
Effects of reintroducing deleted genes on motility and flagella formation in the Mot strain. a, Motility assays on MRS soft agar (0.25%) and flagella observation of recombinant strains. The background strain for all constructs is the Mot strain. Arrows indicate flagella. b, Surface protein profiles of recombinant strains. Arrows indicate the expected size of flagellins. c, Detection of flagellin production in recombinant strains by western blot using anti-FliC1/2 antibodies. Data are representative of three independent experiments.

To investigate whether any of the genes within the deleted genomic region identified by genome sequencing could suppress motility, six genes—including five predicted transcriptional regulators (DegV (NQ504_01055), ROK (NQ504_01140), GntR1 (NQ504_01100), GntR2 (NQ504_01165), RRTF (NQ504_01035)) and one ATP-binding protein (ATPbd (NQ504_01040))—were individually cloned and reintroduced into the Mot strain via conjugation. Each gene was PCR-amplified and inserted into the plasmid pGMβ1, which was then transformed into *Lactococcus lactis* as an intermediate host. However, no transformants were obtained for GntR1, possibly due to a toxic effect of this gene on the host strain. For the remaining constructs, successful transformation into *L. lactis* was followed by conjugative transfer of the plasmids into *L. ruminis* (Mot). The presence of the plasmid and the expression of the introduced genes in the resulting *L. ruminis* transconjugants were confirmed by colony PCR and RT-PCR (Fig. S6). Each recombinant *L. ruminis* strain was then subjected to motility assays using soft agar and examined for flagellum formation. As a result, only the strain complemented with RRTF exhibited a non-motile phenotype, accompanied by the loss of flagella formation and flagellin production (Figure 6ab).

### Pilus formation, adhesion, and Acid/bile tolerance in the RRTF-complemented strain

**Figure 7.**
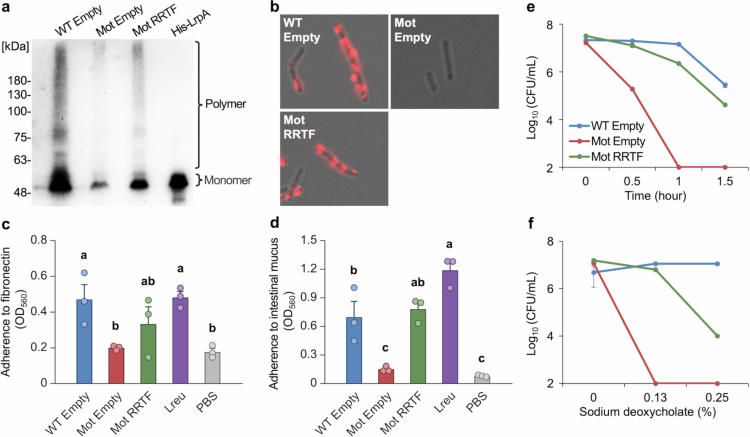
Restoration of pilus formation, adhesion, and acid/bile tolerance in RRTF (FpsR)-expressing Mot strains. WT empty, Mot empty, and Mot RRTF indicate *L. ruminis* strains harboring pGMβ1-based plasmids: an empty vector in WT and Mot strains, and an RRTF expression construct in the Mot strain. a, Detection of pilus proteins by western blot using LrpA-specific antibodies. b, Immunofluorescence staining showing pilus formation and localization using LrpA-specific antibodies. Magnification: × 1000, excitation wavelengths:470/40, emission wavelengths:525/50. c, d, Adhesion assays to fibronectin (c) and mouse intestinal mucus (d). *n* = 3, mean ± SE; One-way ANOVA with Tukey multiple comparisons test; no significant differences between groups among the same letter (*P* ≥ 0.05). e, f, Acid tolerance (e) and bile salt tolerance (f) assays. *n* = 3, mean ± SE.

Analysis of the RRTF-complemented strain revealed restoration of pilin production and formation of pilus polymers (Figure 7a). Fluorescence immunostaining further confirmed that pili were formed on the bacterial surface in the RRTF-complemented strain (Figure 7b). Adhesion assays demonstrated that the adhesive capacity, which had been lost in the Mot strain, showed a tendency to recover upon reintroduction of RRTF when tested with fibronectin (Figure 7c), and was significantly restored in adhesion to mouse intestinal mucus (Figure 7d). Additionally, we examined acid and bile tolerance in the RRTF-complemented strain. Both acid and bile resistance, which were diminished in the Mot strain, showed a trend toward recovery following RRTF complementation (Figure 7ef).

## Discussion

This study provides the first evidence that a human-derived strain of *Lactobacillus ruminis* is capable of full motility through flagellum formation. The motility-acquired mutant exhibited a shorter cell length compared to the wild-type strain. More than 50 genes are required for the assembly and function of bacterial flagella.^25^ In addition, it has been estimated that flagellar rotation and biosynthesis consume approximately 0.1% and 2% of the total cellular energy budget, respectively.^26^ It is likely that the substantial energy cost associated with flagellum formation and motility imposes a metabolic burden, resulting in reduced biomass despite maintenance of overall cell numbers. The motility-acquired mutant strain exhibited chemotaxis, at least in response to acidic conditions. It has been reported that gut-associated bacteria display chemotactic responses toward a variety of intestinal compounds. For example, *Helicobacter pylori* exhibits chemotaxis toward urea, and *Escherichia coli* has been shown to respond to short-chain fatty acids and polyamines.^27,28^ In *Lactobacillus agilis* strains isolated from the chicken intestine, chemotaxis toward short-chain fatty acids and bile acids has been documented.^13^ In this study, we identified an acid-avoidance chemotactic response in a human-derived motile *L. ruminis* strain. This finding may reflect an adaptive behavior to acidic environments in the intestine, which can result from host-derived or microbiota-derived acid production.

The acquisition of motility in this mutant was caused by the loss of a specific genomic region. This deleted region was flanked by genes annotated as IS256 family transposases, raising the possibility that these elements mediated the deletion. The gene expression profile of the mutant differed substantially from that of the wild-type strain. Since the affected locus contained multiple transcription factor genes, it is likely that the loss of these regulatory elements affected the expression of a wide range of genes, extending beyond motility-related genes.

Complementation experiments with individual genes from the deleted region identified RRTF as a gene responsible for suppressing motility in the wild-type strain. RRTF is a component of a typical two-component regulatory system, generally composed of a histidine kinase and a response regulator. The RRTF protein contains a signal transduction response regulator receiver domain as well as an OmpR/PhoB-type DNA-binding domain. Two-component systems of this kind are known to regulate motility in Gram-negative bacteria.^29-31^ The gene located immediately downstream of RRTF, annotated as an ATP-binding protein, contains both a HAMP domain and a histidine kinase domain. It is likely activated by unknown extracellular signals and may regulate RRTF activity. In the RRTF-complemented strain, this putative counterpart was not restored, suggesting that phosphorylation-based regulation may not be functioning normally. Nevertheless, motility suppression was still observed, suggesting that RRTF might inhibit motility in its unphosphorylated state and promote it upon phosphorylation. Alternatively, RRTF may be phosphorylated via a different pathway and function as a negative regulator of motility in its phosphorylated form. Interestingly, RRTF appeared not only to suppress flagella formation but also to promote pili expression. Two-component systems involved in pilus regulation have been reported in *Streptococcus* species, including CsrRS and TCS08.^32,33^ Furthermore, systems that switch between flagellar suppression and pilus expression have been described in *Pseudomonas aeruginosa*, where second messengers such as cyclic-di-GMP and cAMP mediate this regulatory switch.^34^ While the specific signals that regulate this switch in *L. ruminis* remain unidentified, our findings suggest the existence of a flagellum–pilus regulatory switch in this species, with RRTF playing a central role. Based on these findings, we propose to designate this gene as *fpsR* (flagellation-piliation switchover regulator).

The motility-acquired mutant was more susceptible to both acidic conditions and bile acids compared to the wild-type strain. In *L. ruminis*, several genes have been reported to contribute to acid tolerance, including *clpB* (NQ504_05055), the molecular chaperone *grpE* (NQ504_05100), and *queA* (NQ504_07895).^35^ In the present RNA-seq analysis, no substantial differences in the expression levels of these genes were observed between the strains under standard conditions. However, it is possible that under stress conditions, expression levels may diverge. Alternatively, flagellum formation itself may compromise resistance to acid and bile. Since flagellar rotation is driven by the proton motive force, maintaining this gradient under acidic conditions likely imposes a significant energetic burden on the cell. Intracellular pH homeostasis is critical for acid tolerance, and motility may interfere with this regulatory mechanism. Regarding bile acid tolerance, the expression level of a choloylglycine hydrolase family protein was found to be reduced in the motility-acquired mutant, which may partially account for its increased bile sensitivity. Interestingly, in the *fpsR* (RRTF)-complemented strain, both acid and bile acid tolerance were restored. Although further investigation is needed, these findings suggest that *fpsR* may also regulate stress response pathways.

The flagellar antigen of human-derived *L. ruminis* exhibited proinflammatory activity comparable to that of strains derived from other animal hosts, with its potency estimated to be on par with that of certain pathogenic bacteria. In our previous study, we reported that the flagellin of the closely related species *L. agilis* elicited only minimal inflammatory responses, and we proposed that such low immunostimulatory activity is an important feature for maintaining a non-inflammatory relationship with the host as a gut commensal bacterium.^14,15^ In contrast, the flagellar antigen of *L. ruminis* demonstrated significantly stronger inflammatory activity than that of *L. agilis*. Therefore, if large quantities of *L. ruminis* flagellin were produced within the intestinal environment, it could potentially contribute to intestinal inflammation and adverse host outcomes. Indeed, previous studies have reported a positive correlation between the abundance of *L. ruminis* in the human gut and the incidence of type II diabetes and ischemic stroke.^36,37^ However, in the present study, the motile *L. ruminis* mutant strain was unable to colonize the animal intestine, and no evidence of excessive flagellin production or inflammation was observed in the gut, even in animals in which the wild-type, pilus-forming strain had established dominance. Although the experimental conditions involved a short-term exposure in a non-natural host environment, and definitive conclusions cannot yet be drawn, our results suggest that *L. ruminis* strains residing in the human gut are unlikely to trigger inflammation via flagellin production. Notably, Neville et al. reported the presence of *L. ruminis* cells displaying partial motility (tumbling) following passage through the murine gastrointestinal tract.^24^ Given that the wild-type strain has the potential to acquire motility through deletion or mutation of *fpsR*, it is plausible that some motility mutants could arise in the gut environment. However, such motility-acquired variants would likely lose the ability to form pili and, consequently, lose their capacity for stable colonization. Thus, it is unlikely that they would persist or become dominant in the gut microbial ecosystem.

The primary factor preventing the motility-acquired mutant from colonizing the murine intestine appears to be the loss of pilus formation. Initially, this was thought to be a collateral effect of the large genomic deletion; however, our findings suggest that flagellum and pilus formation represent two distinct physiological phases, motility and colonization, which appear to represent mutually exclusive states regulated by *fpsR*. The motility-acquired mutant, lacking this regulatory switch, was unable to transition from the motile to the adhesive phase within the gut environment, thereby failing to establish colonization. This naturally raises the question: what is the ecological significance of flagellum formation? Given the high energetic cost of flagellar synthesis and rotation, flagella would likely be eliminated from the genome if they were unnecessary. Indeed, most lactic acid bacteria completely lack the genes required for flagellar assembly. Nevertheless, *L. ruminis* has retained multiple chemotaxis receptors and a full complement of motility-related genes, indicating a potential ecological role for motility. Although our study does not directly answer this question, possibilities are that motility may be advantageous in environments outside the host gut—such as during environmental survival after fecal excretion—or during mother-to-infant or horizontal microbial transmission, when the ability to reach a suitable niche could be critical. Motility might also play a role in emergency responses (“scramble mode”) under abnormal conditions such as diarrhea, where intestinal contents become more fluid. Another intriguing question concerns the observation that *L. ruminis* strains isolated from other animals frequently display motility under standard laboratory conditions. It is possible that even in these animal hosts, the bacteria exist primarily in a pilus-expressing, colonization phase in vivo. The apparent motility observed in vitro might simply reflect a less stringent suppression of motility gene expression compared to human-derived strains. These possibilities warrant further investigation in future studies.

To date, studies on bacterial motility in the gut environment have primarily focused on its association with pathogenicity. However, certain commensal and symbiotic bacteria—such as members of the genera *Ligilactobacillus* and *Roseburia*—also possess the ability to form flagella.^38,39^ The ecological roles of these flagellated commensals and their impacts on host–microbe interactions remain largely unexplored. This study provides a new framework for understanding how flagellated commensals regulate surface structures and host interaction.

## Animal study approval statement

All animal procedures were conducted in accordance with institutional guidelines and approved by the Animal Experiment Committee of Tokyo University of Agriculture (Approval No. 240097).

## Supplementary Material

Supplementary Material Revised Suppl_Clean ver

Supplementary Material SuptInfo_S1

Supplementary Material SM1mp4

Supplementary Material SM2mp4

## Data Availability

DNA sequencing data of the Mot strain have been deposited in the DDBJ Sequence Read Archive (DRA) under the BioProject identifier PRJDB35753 (accession number DRR727981). RNA-seq data comparing the WT and Mot strains have been deposited under the same BioProject ID PRJDB35753 (accession numbers DRR727982 and DRR727983).

## References

[1] O’Connell Motherway M, Zomer A, Leahy SC, Reunanen J, Bottacini F, Claesson MJ, O'Connell Motherway M, O'Brien F, Flynn K, Casey PG, et al. Functional genome analysis of *Bifidobacterium breve* UCC2003 reveals type IVb tight adherence (Tad) pili as an essential and conserved host-colonization factor. Pro Natl Acad Sci. 2011;108(27):11217–11222. doi: 10.1073/pnas.1105380108. PMC3131351 21690406

[2] Turroni F, Serafini F, Foroni E, Duranti S, O’Connell Motherway M, Taverniti V, Mangifesta M, Milani C, Viappiani A, Roversi T, et al. Role of sortase-dependent pili of *Bifidobacterium bifidum* PRL2010 in modulating bacterium–host interactions. Proc Natl Acad Sci. 2013;110(27):11151–11156. doi: 10.1073/pnas.1303897110. 23776216 PMC3703987

[3] Rintahaka J, Yu X, Kant R, Palva A, von Ossowski I. Phenotypical analysis of the lactobacillus rhamnosus GG fimbrial spaFED operon: surface expression and functional characterization of Recombinant SpaFED pili in lactococcus lactis. PLoS One. 2014;9(11):e113922. doi: 10.1371/journal.pone.0113922. 25415357 PMC4240662

[4] Leccese Terraf MC, Mendoza LM, Juárez Tomás MS, Silva C, Nader-Macías MEF. Phenotypic surface properties (aggregation, adhesion and biofilm formation) and presence of related genes in beneficial vaginal *lactobacilli*. J Appl Microbiol. 2014;117(6):1761–1772. doi: 10.1111/jam.12642. 25195810

[5] Yu X, Jaatinen A, Rintahaka J, Hynönen U, Lyytinen O, Kant R, Åvall-Jääskeläinen S, von Ossowski I, Palva A, Freitag NE. Human gut-commensalic lactobacillus ruminis ATCC 25644 displays sortase-assembled surface piliation: phenotypic characterization of its fimbrial operon through in silico predictive analysis and recombinant expression in lactococcus lactis. PLoS One. 2015;10(12):e0145718. doi: 10.1371/journal.pone.0145718. 26709916 PMC4692528

[6] Grant CC, Konkel ME, Cieplak W, Tompkins LS. Role of flagella in adherence, internalization, and translocation of Campylobacter jejuni in nonpolarized and polarized epithelial cell cultures. Infect Immun. 1993;61(5):1764–1771. doi: 10.1128/iai.61.5.1764-1771.1993. 8478066 PMC280763

[7] Liu SL, Ezaki T, Miura H, Matsui K, Yabuuchi E. Intact motility as a Salmonella typhi invasion-related factor. Infect Immun. 1988;56(8):1967–1973. doi: 10.1128/iai.56.8.1967-1973.1988. 2840399 PMC259509

[8] Mobley HL, Belas R, Lockatell V, Chippendale G, Trifillis AL, Johnson DE, Warren JW. Construction of a flagellum-negative mutant of Proteus mirabilis: effect on internalization by human renal epithelial cells and virulence in a mouse model of ascending urinary tract infection. Infect Immun. 1996;64(12):5332–5340. doi: 10.1128/iai.64.12.5332-5340.1996. 8945585 PMC174527

[9] Tasteyre A, Barc MC, Collignon A, Boureau H, Karjalainen T. Role of FliC and FliD flagellar proteins of *Clostridium difficile* in adherence and gut colonization. Infect Immun. 2001;69(12):7937–7940. doi: 10.1128/IAI.69.12.7937-7940.2001. 11705981 PMC98895

[10] Ottemann KM, Lowenthal AC. *Helicobacter pylori* uses motility for initial colonization and to attain robust infection. Infect Immun. 2002;70(4):1984–1990. doi: 10.1128/IAI.70.4.1984-1990.2002. 11895962 PMC127824

[11] Zhou B, Szymanski CM, Baylink A. Bacterial chemotaxis in human diseases. Trends Microbiol. 2023;31(5):453–467. doi: 10.1016/j.tim.2022.10.007. 36411201 PMC11238666

[12] Kajikawa A, Suzuki S, Igimi S. The impact of motility on the localization of Lactobacillus agilis in the murine gastrointestinal tract. BMC Microbiol. 2018;18(1):68. doi: 10.1186/s12866-018-1219-3. 29996774 PMC6042280

[13] Suzuki S, Yokota K, Igimi S, Kajikawa A. Negative chemotaxis of Ligilactobacillus agilis BKN88 against gut-derived substances. Sci Rep. 2023;13(1):15632. doi: 10.1038/s41598-023-42840-5. 37730901 PMC10511705

[14] Eguchi N, Suzuki S, Yokota K, Igimi S, Kajikawa A. Ligilactobacillus agilis BKN88 possesses thermo-/acid-stable heteropolymeric flagellar filaments. Microbiology (N Y). 2021;167(3). doi: 10.1099/mic.0.001020. 33502302

[15] Kajikawa A, Midorikawa E, Masuda K, Kondo K, Irisawa T, Igimi S, Okada S. Characterization of flagellins isolated from a highly motile strain of Lactobacillus agilis. BMC Microbiol. 2016;16(1):49. doi: 10.1186/s12866-016-0667-x. 27001290 PMC4802830

[16] Clasen SJ, Bell MEW, Borbón A, Lee DH, Henseler ZM, de la Cuesta-Zuluaga J, Parys K, Zou J, Wang Y, Altmannova V, et al. Silent recognition of flagellins from human gut commensal bacteria by Toll-like receptor 5. Sci Immunol. 2023;8(79). doi: 10.1126/sciimmunol.abq7001. 36608151

[17] Kang JY, Lee DK, Ha NJ, Shin HS. Antiviral effects of Lactobacillus ruminis SPM0211 and Bifidobacterium longum SPM1205 and SPM1206 on rotavirus-infected Caco-2 cells and a neonatal mouse model. J Microbiol. 2015;53(11):796–803. doi: 10.1007/s12275-015-5302-2. 26502964 PMC7090939

[18] Taweechotipatr M, Iyer C, Spinler JK, Versalovic J, Tumwasorn S. *Lactobacillus saerimneri* and *Lactobacillus ruminis*: novel human-derived probiotic strains with immunomodulatory activities. FEMS Microbiol Lett. 2009;293(1):65–72. doi: 10.1111/j.1574-6968.2009.01506.x. 19222575 PMC4105522

[19] Yang B, Li M, Wang S, Ross RP, Stanton C, Zhao J, Zhang H, Chen W. Lactobacillus ruminis alleviates DSS-induced colitis by inflammatory cytokines and gut microbiota modulation. Foods. 2021;10(6):1349. doi: 10.3390/foods10061349. 34208038 PMC8230674

[20] Yu X, Åvall-Jääskeläinen S, Koort J, Lindholm A, Rintahaka J, von Ossowski I, Palva A, Hynönen U. A comparative characterization of different host-sourced lactobacillus ruminis strains and their adhesive, inhibitory, and immunomodulating functions. Front Microbiol. 2017;8:657. doi: 10.3389/fmicb.2017.00657. 28450859 PMC5390032

[21] O’ Donnell MM, Harris HMB, Lynch DB, Ross RP, O’Toole PW. Lactobacillus ruminis strains cluster according to their mammalian gut source. BMC Microbiol. 2015;15(1):80. doi: 10.1186/s12866-015-0403-y. 25879663 PMC4393605

[22] SHARPE ME, LATHAM MJ, GARVIE EI, ZIRNGIBL J, KANDLER O. Two new species of lactobacillus isolated from the bovine rumen, lactobacillus ruminis sp.nov. and lactobacillus vitulinus sp.nov. J Gen Microbiol. 1973;77(1):37–49. doi: 10.1099/00221287-77-1-37. 4723944

[23] Ghosh TS, Arnoux J, O’Toole PW. Metagenomic analysis reveals distinct patterns of gut lactobacillus prevalence, abundance, and geographical variation in health and disease. Gut Microbes. 2020;12(1):1822729. doi: 10.1080/19490976.2020.1822729. 32985923 PMC7524322

[24] Neville BA, Forde BM, Claesson MJ, Darby T, Coghlan A, Nally K, Ross RP, O’Toole PW, Planet PJ. Characterization of pro-inflammatory flagellin proteins produced by lactobacillus ruminis and related motile lactobacilli. PLoS One. 2012;7(7):e40592. doi: 10.1371/journal.pone.0040592. 22808200 PMC3393694

[25] Macnab RM. How bacteria assemble flagella. Annu Rev Microbiol. 2003;57(1):77–100. doi: 10.1146/annurev.micro.57.030502.090832. 12730325

[26] Macnab R. Flagella and motility. ASM Press; 1996; p. 123–145.

[27] Tobe T, Nakanishi N, Sugimoto N. Activation of motility by sensing short-chain fatty acids via two steps in a flagellar gene regulatory cascade in enterohemorrhagic *Escherichia coli*. Infect Immun. 2011;79(3):1016–1024. doi: 10.1128/IAI.00927-10. 21149585 PMC3067497

[28] Lopes JG, Sourjik V. Chemotaxis of *Escherichia coli* to major hormones and polyamines present in human gut. ISME J. 2018;12(11):2736–2747. doi: 10.1038/s41396-018-0227-5. 29995838 PMC6194112

[29] Tipton KA, Rather PN. An *ompR-envZ* two-component system ortholog regulates phase variation, osmotic tolerance, motility, and virulence in acinetobacter baumannii strain AB5075. J Bacteriol. 2017;199(3). doi: 10.1128/JB.00705-16. PMC5237114 27872182

[30] Raczkowska A, Skorek K, Bielecki J, Brzostek K. OmpR controls Yersinia enterocolitica motility by positive regulation of flhDC expression. Antonie Van Leeuwenhoek. 2011;99(2):381–394. doi: 10.1007/s10482-010-9503-8. 20830609 PMC3032193

[31] Huo X, Du C, Huang H, Gu H, Dong X, Hu Y. TCS response regulator OmpR plays a major role in stress resistance, antibiotic resistance, motility, and virulence in Edwardsiella piscicida. Aquaculture. 2022;559:738441. doi: 10.1016/j.aquaculture.2022.738441.

[32] Song XM, Connor W, Hokamp K, Babiuk LA, Potter AA. The growth phase-dependent regulation of the pilus locus genes by two-component system TCS08 in Streptococcus pneumoniae. Microb Pathog. 2009;46(1):28–35. doi: 10.1016/j.micpath.2008.10.006. 18983906

[33] Jiang S, Park SE, Yadav P, Paoletti LC, Wessels MR. Regulation and Function of Pilus Island 1 in Group B Streptococcus. J Bacteriol. 2012;194(10):2479–2490. doi: 10.1128/JB.00202-12. 22408160 PMC3347183

[34] Kazmierczak BI, Schniederberend M, Jain R. Cross-regulation of Pseudomonas motility systems: the intimate relationship between flagella, pili and virulence. Curr Opin Microbiol. 2015;28:78–82. doi: 10.1016/j.mib.2015.07.017. 26476804 PMC4688086

[35] Park S, Park MA, Jang HJ, Kim DH, Kim Y. Complete genome sequence of potential probiotic Ligilactobacillus ruminis CACC881 isolated from swine. J Anim Sci Technol. 2024. doi: 10.5187/jast.2024.e50. PMC12715446 41426210

[36] Yamashiro K, Tanaka R, Urabe T, Ueno Y, Yamashiro Y, Nomoto K, Takahashi T, Tsuji H, Asahara T, Hattori N, et al. Gut dysbiosis is associated with metabolism and systemic inflammation in patients with ischemic stroke. PLoS One. 2017;12(2):e0171521. doi: 10.1371/journal.pone.0171521. 28166278 PMC5293236

[37] Carrizales-Sánchez AK, Tamez-Rivera O, García-Gamboa R, García-Cayuela T, Rodríguez-Gutiérrez NA, Elizondo-Montemayor L, García-Rivas G, Pacheco A, Hernández-Brenes C, Senés-Guerrero C. Gut microbial composition and functionality of school-age Mexican population with metabolic syndrome and type-2 diabetes mellitus using shotgun metagenomic sequencing. Front Pediatr. 2023;11:1193832. doi: 10.3389/fped.2023.1193832. 37342535 PMC10277889

[38] Cousin FJ, Lynch SM, Harris HMB, McCann A, Lynch DB, Neville BA, Irisawa T, Okada S, Endo A, O'Toole PW, et al. Detection and genomic characterization of motility in lactobacillus curvatus: confirmation of motility in a species outside the lactobacillus salivarius clade. Appl Environ Microbiol. 2015;81(4):1297–1308. doi: 10.1128/AEM.03594-14. 25501479 PMC4309690

[39] Nie K, Ma K, Luo W, Shen Z, Yang Z, Xiao M, Tong T, Wang X. Roseburia intestinalis: a beneficial Gut organism from the discoveries in Genus and Species. Front Cell Infect Microbiol. 2021;11. doi: 10.3389/fcimb.2021.757718. PMC8647967 34881193

[40] Liong MT, Shah NP. Acid and bile tolerance and cholesterol removal ability of lactobacilli strains. J Dairy Sci. 2005;88(1):55–66. doi: 10.3168/jds.S0022-0302(05)72662-X. 15591367

[41] Pfeiler EA, Azcarate-Peril MA, Klaenhammer TR. Characterization of a novel bile-inducible operon encoding a two-component regulatory system in *Lactobacillus acidophilus*. J Bacteriol. 2007;189(13):4624–4634. doi: 10.1128/JB.00337-07. 17449631 PMC1913432

[42] Gaucher F, Bonnassie S, Rabah H, Marchand P, Blanc P, Jeantet R, Jan G. Review: adaptation of beneficial propionibacteria, lactobacilli, and bifidobacteria improves tolerance toward technological and digestive stresses. Front Microbiol. 2019;10:841. doi: 10.3389/fmicb.2019.00841. 31068918 PMC6491719

[43] Andersen-Nissen E, Smith KD, Bonneau R, Strong RK, Aderem A. A conserved surface on Toll-like receptor 5 recognizes bacterial flagellin. J Exp Med. 2007;204(2):393–403. doi: 10.1084/jem.20061400. 17283206 PMC2118731

[44] Smith KD, Andersen-Nissen E, Hayashi F, Strobe K, Bergman MA, Barrett SLR, Cookson BT, Aderem A. Toll-like receptor 5 recognizes a conserved site on flagellin required for protofilament formation and bacterial motility. Nat Immunol. 2003;4(12):1247–1253. doi: 10.1038/ni1011. 14625549

[45] Kajikawa A, Eguchi N, Suzuki S. Immunogenic modification of ligilactobacillus agilis by specific amino acid substitution of flagellin. Appl Environ Microbiol. 2022;88(20):e01277-22. doi: 10.1128/aem.01277-22. 36173204 PMC9599256

[46] Twine SM, Paul CJ, Vinogradov E, McNally DJ, Brisson J, Mullen JA, McMullin DR, Jarrell HC, Austin JW, Kelly JF, et al. Flagellar glycosylation in *Clostridium botulinum*. FEBS J. 2008;275(17):4428–4444. doi: 10.1111/j.1742-4658.2008.06589.x. 18671733

[47] Schirm M, Kalmokoff M, Aubry A, Thibault P, Sandoz M, Logan SM. Flagellin from *Listeria monocytogenes* Is Glycosylated with β-O-Linked *N* -Acetylglucosamine. J Bacteriol. 2004;186(20):6721–6727. doi: 10.1128/JB.186.20.6721-6727.2004. 15466023 PMC522210

[48] Vargas García CE, Petrova M, Claes IJJ, De Boeck I, Verhoeven TLA, Dilissen E, von Ossowski I, Palva A, Bullens DM, Vanderleyden J, et al. Piliation of lactobacillus rhamnosus GG promotes adhesion, phagocytosis, and cytokine modulation in macrophages. Appl Environ Microbiol. 2015;81(6):2050–2062. doi: 10.1128/AEM.03949-14. 25576613 PMC4345371

[49] Claes IJJ, Schoofs G, Regulski K, Courtin P, Chapot-Chartier MP, Rolain T, Hols P, von Ossowski I, Reunanen J, de Vos WM, et al. Genetic and biochemical characterization of the cell wall hydrolase activity of the major secreted protein of lactobacillus rhamnosus GG. PLoS One. 2012;7(2):e31588. doi: 10.1371/journal.pone.0031588. 22359601 PMC3281093

[50] Chang C, Huang IH, Hendrickx APA, Ton-That H. Visualization of gram-positive bacterial pili. Methods Mol Biol. 2013;966:77–95. 23299729 10.1007/978-1-62703-245-2_5

[51] Reunanen J, von Ossowski I, Hendrickx APA, Palva A, de Vos WM. Characterization of the SpaCBA Pilus Fibers in the Probiotic Lactobacillus rhamnosus GG. Appl Environ Microbiol. 2012;78(7):2337–2344. doi: 10.1128/AEM.07047-11. 22247175 PMC3302623

[52] Tierney JB, Matthews E, Carrington SD, Mulcahy G. Interaction of Eimeria tenella with Intestinal Mucin In Vitro. Source: The Journal of Parasitology. 2007;93:634–638. doi: 10.1645/GE-1066R.1. 17626356

[53] Alemka A, Whelan S, Gough R, Clyne M, Gallagher ME, Carrington SD, Bourke B. Purified chicken intestinal mucin attenuates Campylobacter jejuni pathogenicity in vitro. J Med Microbiol. 2010;59(8):898–903. doi: 10.1099/jmm.0.019315-0. 20466838

[54] MIYOSHI Y, OKADA S, UCHIMURA T, SATOH E. A mucus adhesion promoting protein, mapa, mediates the adhesion of *Lactobacillus reuteri* to Caco-2 human intestinal epithelial cells. Biosci Biotechnol Biochem. 2006;70(7):1622–1628. doi: 10.1271/bbb.50688. 16861796

[55] Plant L, Lam C, Conway PL, O’Riordan K. Gastrointestinal microbial community shifts observed following oral administration of a Lactobacillus fermentum strain to mice. FEMS Microbiol Ecol. 2003;43(2):133–140. doi: 10.1111/j.1574-6941.2003.tb01052.x. 19719673

[56] Ubeda C, Bucci V, Caballero S, Djukovic A, Toussaint NC, Equinda M, Lipuma L, Ling L, Gobourne A, No D, et al. Intestinal microbiota containing barnesiella species cures vancomycin-resistant enterococcus faecium colonization. Infect Immun. 2013;81(3):965–973. doi: 10.1128/IAI.01197-12. 23319552 PMC3584866

[57] Tan J, Gong J, Liu F, Li B, Li Z, You J, He J, Wu S. Evaluation of an antibiotic cocktail for fecal microbiota transplantation in mouse. Front Nutr. 2022;9:918098. doi: 10.3389/fnut.2022.918098. 35719145 PMC9204140

[58] Duong T, Miller MJ, Barrangou R, Azcarate‐Peril MA, Klaenhammer TR. Construction of vectors for inducible and constitutive gene expression in *Lactobacillus*. Microb Biotechnol. 2011;4(3):357–367. doi: 10.1111/j.1751-7915.2010.00200.x. 21375708 PMC3818994

[59] Iwamoto D, Ishizaki M, Miura T, Sasaki Y. Novel shuttle vector pGMβ1 for conjugative chromosomal manipulation of *Lactobacillus delbrueckii* subsp. *bulgaricus*. Biosci Microbiota Food Health. 2022;41(1):20–29. 35036250 10.12938/bmfh.2021-014 PMC8727053

[60] Sasaki Y, Taketomo N, Sasaki T. Factors affecting transfer frequency of pAM beta 1 from Streptococcus faecalis to Lactobacillus plantarum. J Bacteriol. 1988;170(12):5939–5942. doi: 10.1128/jb.170.12.5939-5942.1988. 3142863 PMC211709

[61] de Ruyter PG, Kuipers OP, Beerthuyzen MM, van Alen-Boerrigter I, de Vos WM. Functional analysis of promoters in the nisin gene cluster of Lactococcus lactis. J Bacteriol. 1996;178(12):3434–3439. doi: 10.1128/jb.178.12.3434-3439.1996. 8655538 PMC178110

[62] Boot HJ, Kolen CP, van Noort JM, Pouwels PH. S-layer protein of Lactobacillus acidophilus ATCC 4356: purification, expression in Escherichia coli, and nucleotide sequence of the corresponding gene. J Bacteriol. 1993;175(19):6089–6096. 8407780 10.1128/jb.175.19.6089-6096.1993 PMC206701

[63] Bécavin C, Bouchier C, Lechat P, Archambaud C, Creno S, Gouin E, Wu Z, Kühbacher A, Brisse S, Pucciarelli MG, et al. Comparison of widely used listeria monocytogenes strains EGD, 10403S, and EGD-e highlights genomic differences underlying variations in pathogenicity. mBio. 2014;5(2): e00969-14. doi: 10.1128/mbio.00969-14. 24667708 PMC3977354

