# Supplementary material for: The response regulator FpsR controls the flagella–pili transition and mucosal colonization in Ligilactobacillus ruminis
Source: Gut Microbes. 2025 Dec 4;17(1):2596807. doi: 10.1080/19490976.2025.2596807 (PMC12688256; doi:10.1080/19490976.2025.2596807)
Supplement: Supplementary Material — Revised Suppl_Clean ver [file KGMI_A_2596807_SM2256.docx]

**Supplemental Information**

**The Response Regulator FpsR Controls the Flagella–Pili Transition and Mucosal Colonization in *Ligilactobacillus ruminis***

Aya Misaki^1^, Shunya Suzuki^2^, Shintaro Maeno^3^, Akihito Endo^4^, Yasuko Sasaki^5^, Gen Enomoto^1^, 　Kenji Yokota^1^, and Akinobu Kajikawa^1^*

^1^ Department of Agricultural Chemistry, Tokyo University of Agriculture, Tokyo 156-0054, Japan ^2^ Biomanufacturing and Process Research Center, National Institute of Advanced Industrial Science and Technology, Ibaraki 305-8566, Japan ^3^ Research Center for Thermotolerant Microbial Resources, Yamaguchi University, Yamaguchi 753-8515, Japan

^4^ Department of Nutritional Science and Food Safety, Tokyo University of Agriculture, Tokyo 156-0054, Japan

^5^Department of Agricultural Chemistry, Meiji University, Kanagawa 214-8571, Japan

***Corresponding author**

Akinobu Kajikawa

Email: [a3kajika@nodai.ac.jp](mailto:a3kajika@nodai.ac.jp)

**This file includes:**

Figures S1 to S6

Tables S1 to S2

Legends for Movies S1 to S2

Legends for Datasets S1

**Other supporting materials for this manuscript include the following:**

Movies S1 to S2

Supporting Information S1


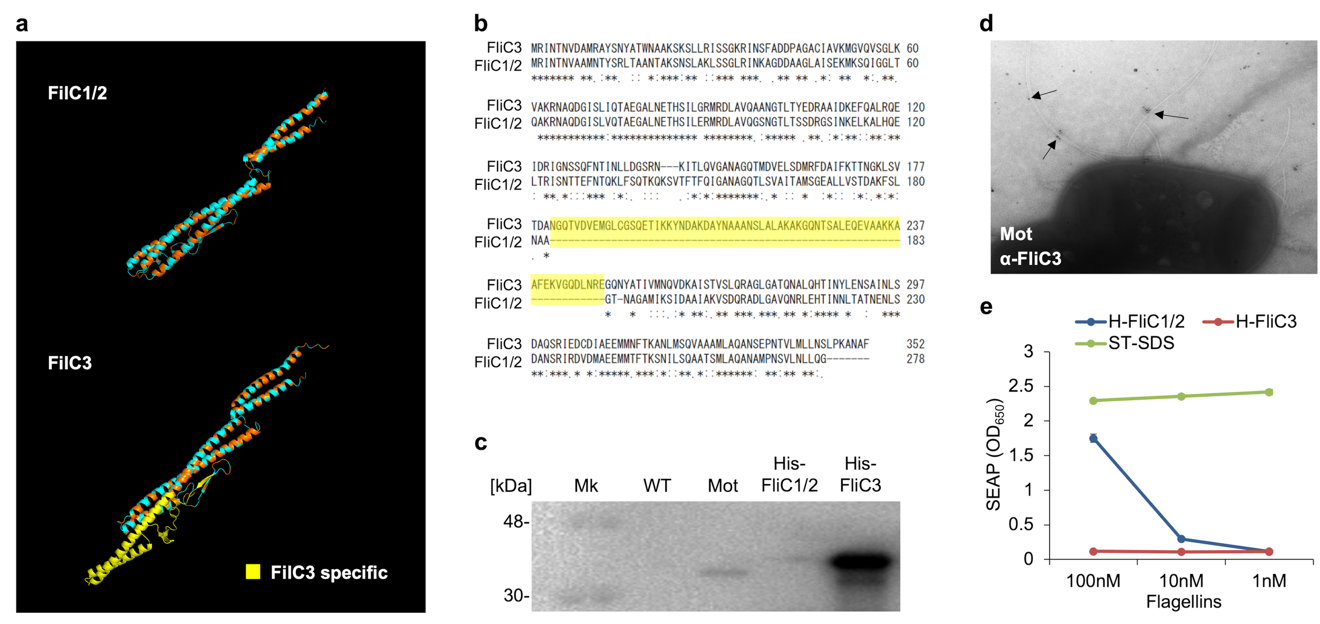


N

C

N

C

Figure S1. Structure prediction and functional analysis of minor flagellin, FliC3. a, Predicted structures of FliC1/2 and FliC3 generated by AlphaFold3 and visualized with PyMOL. N- and C-termini are indicated as N and C, respectively. Orange indicates hydrophobic amino acid residues, light blue indicates hydrophilic amino acid residues, and yellow highlights regions unique to FliC3 with low homology to FliC1/2. b, Sequence alignment of FliC3 and FliC1/2. FliC3-specific sequences are highlighted in yellow. c, Western blot analysis using anti-FliC3 antibodies. d, Immunogold labelling using anti-FliC3 antibodies. e, Reporter gene assays using HEK-Blue hTLR5 cells stimulated with recombinant flagellins (H-FliC1/2, H-FliC3) or with monomerized *Salmonella Typhimurium* flagellin containing the maximum SDS concentration possibly present in H-FliC3 preparations (ST-SDS). n = 3; data are presented as mean ± standard error (SE).


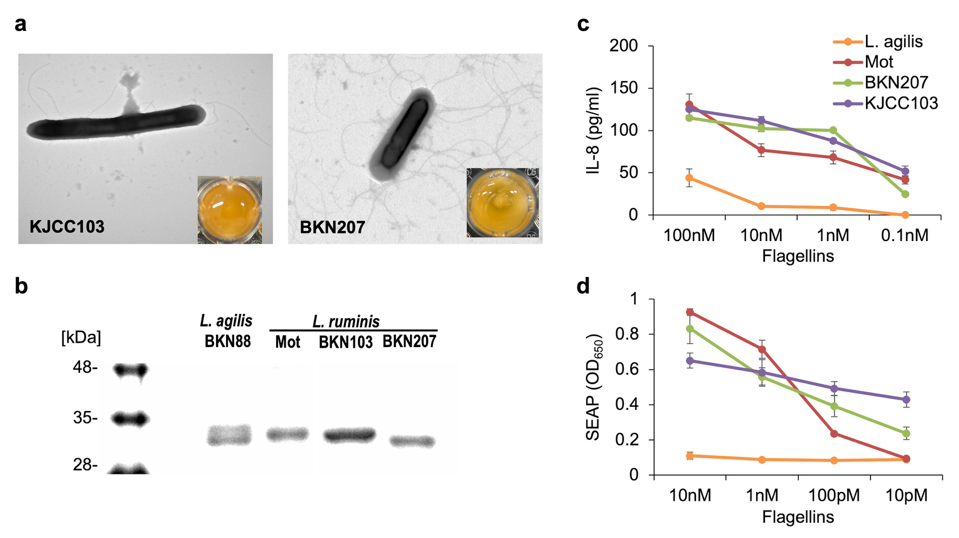


Figure S2. Comparison of inflammation-inducing activity of *L. ruminis* in different host animals. a, Transmission electron microscopy (TEM) of KJCC103 (pig-derived) and BKN207 (horse-derived) strains with negative staining (×6,000). Bottom right: motility assay on soft agar. b, SDS-PAGE of purified flagellins. c, IL-8 production in Caco-2 cells stimulated with purified flagellins. d, Reporter gene assays using HEK-Blue hTLR5 cells stimulated with purified flagellins. n = 3; data are presented as mean ± SE.


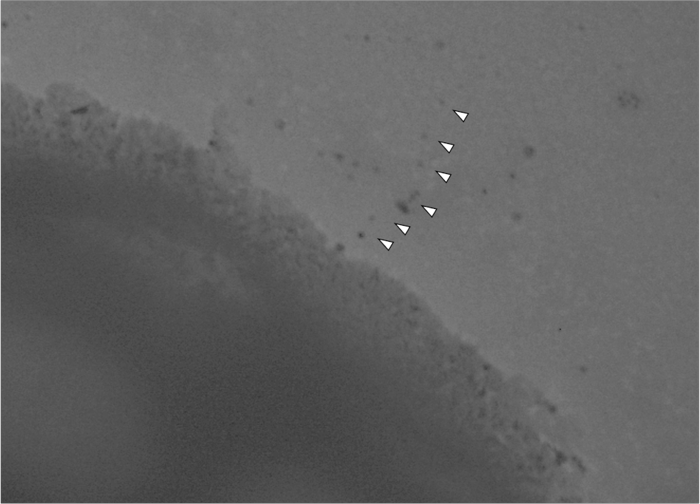


Figure S3. Immunogold labelling of WT using LrpA-specific antibodies. Transmission electron microscopy (TEM) of ATCC 25644 (WT) with pilus-specific immunogold labeling and negative staining.


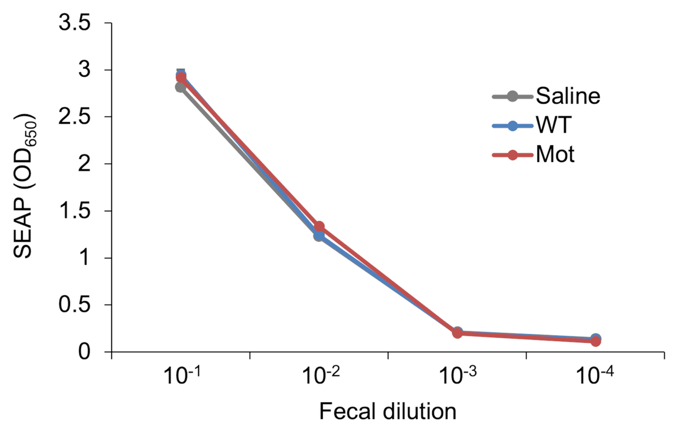


Figure S4. Flagellin activity in fecal extracts. Reporter gene assays using HEK-Blue hTLR5 cells stimulated with diluted fecal extracts. n = 1-2; data are presented as mean value.


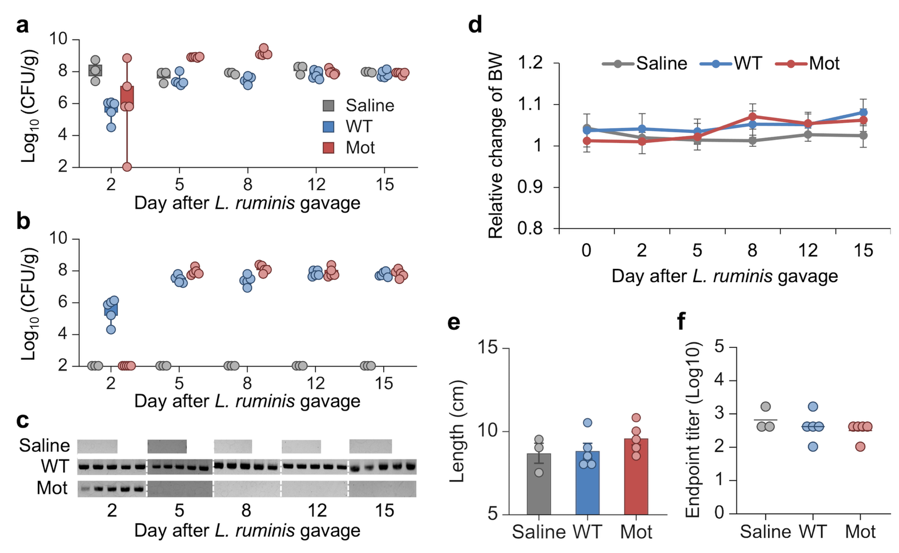


Figure S5. Colonization of *L. ruminis* in the mouse intestine and evaluation of inflammation-related parameters. a, b, Enumeration of viable fecal bacteria over time by anaerobic culture on GAM (a) and MRS agar (b). Each point represents an individual mouse (Saline: 3 mice/ group. WT, Mot:5 mice/group). c, Detection of *L. ruminis* in fecal samples by species-specific PCR. d, Body weight change over time. Mean ± SE e, Comparison of intestinal lengths. Mean ± SE. f, Measurement of *L. ruminis*-specific antibody titers. No significant differences detected by Tukey’s multiple comparison test (d–f).


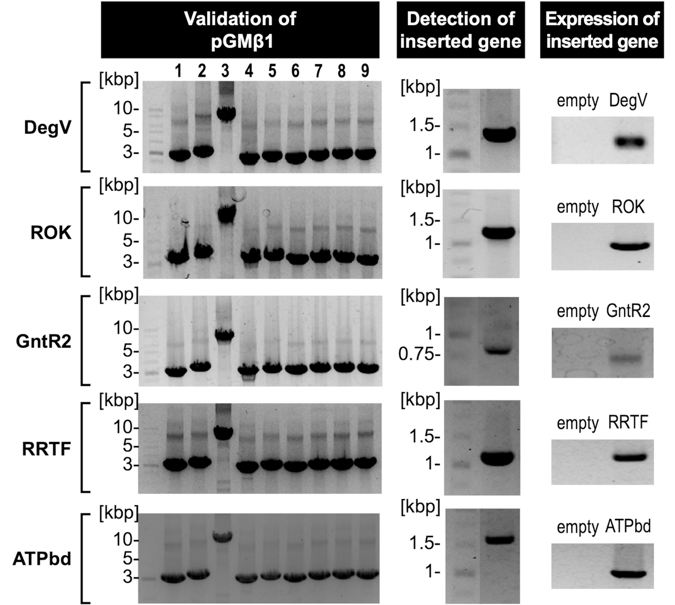


Figure S6. Colony PCR and RT-PCR of *L. ruminis* transconjugants. Colony PCR for validation of pGMβ1 (left) and inserted gene detection (middle). Right: RT-PCR confirming expression of the inserted gene.

**Table S1**. Primer sequences used in this study.

| Primer ID | Sequence |
| --- | --- |
| DOKJ200 | AGAGTTTGATCCTGGCTCAG |
| DOKJ201 | CGGTTACCTTGTTACGACTT |
| DOKJ215 | GCTTTGTGAGCGGATAACAA |
| DOKJ216 | GGATCTATCAACAGGAGTCC |
| DOKJ1398 | GCGCTTAGAATCGCTTTAGGAAAC |
| DOKJ1399 | CGGGTTCTTCAAATATTTCTCCAAG |
| DOKJ1400 | CCCGATTACATGGATTGGATTAGTTC |
| DOKJ1401 | GCACTATCAACACACTCTTAAGTTTG |
| DOKJ1402 | CTTAGAAGCAAACTTAAGAGTGTGTTG |
| DOKJ1403 | GGGTGCTGTTGTTTAAAGGTATC |
| DOKJ1404 | CCCTAATTTTGATGAACTAGCGAAAC |
| DOKJ1405 | CGAGCGAATAGCGAGCAAAATATTAAC |
| DOKJ1406 | GCACCTTTTTCAATTAGACGCTTTG |
| DOKJ1407 | GGCTTGTTTCACTTGATCGCTATTC |
| DOKJ1408 | GGGAGTTAGTTATGAATAGCGATCAAG |
| DOKJ1409 | CGGACTAACGCCGTAAATATCTTC |
| DOKJ1410 | CGGCGTTAGTCCGAAGAAAG |
| DOKJ1411 | GCTTCTACTCCTCTCCTAATTGAATG |
| DOKJ1412 | CCCAAAGAACGACCATTCAATTAG |
| DOKJ1413 | CGCCCTCAAAGACATTAGAGATAG |
| DOKJ1414 | GCTGGTGAGGCTATCTCTAATG |
| DOKJ1415 | GGACTGGATCGTGTTTCCTAAAG |
| DOKJ1964 | cattcgccattcaggctgc |
| DOKJ1965 | cctacaaggggtcccgag |
| DOKJ1991 | aaaGGATCCATGCGAATTAATACGAACGTTGATGC |
| DOKJ1992 | aaaAAGCTTTCAAAACGCATTTGCTTTCGGC |
| DOKJ1993 | aaaGGTACCATGCGTATTAACACAAACGTCGC |
| DOKJ1994 | aaaAAGCTTTTAGCCTTGAAGCAAGTTCAAAACTG |
| DOKJ2029 | aaaGGATCCATGGATGTCGAACTCAGCGAC |
| DOKJ2030 | aaaAAGCTTGGTGCTGATTGCTTTGTCGAC |
| DOKJ2083 | atatGCATGCgcagaaacgtcaacggaaac |
| DOKJ2084 | atatAAGCTTttagatgttttcacgacggttgc |
| DOKJ2131 | ctatagggcgaattgTGCGACAAGTAATAAACTAAACAAAAC |
| DOKJ2151 | GATATTTCATGACTCAAAACAAAGATGAAGC |
| DOKJ2152 | TGAGTCATGAAATATCTCCTTTTAAATTCAATGTTTCATC |
| DOKJ2153 | GagcatgcgacgtcgCTACTTTCCAAAGACGTTAATTTGC |
| DOKJ2154 | GATATTTCATGAACAAAAAACAGGAATCAAACT |
| DOKJ2155 | TTGTTCATGAAATATCTCCTTTTAAATTCAATGTTTCATC |
| DOKJ2156 | GagcatgcgacgtcgCTACGAATTAAGCTTATCGTACCTG |
| DOKJ2178 | GATATTTCATGTCTACAAAATACCAAGTCGTC |
| DOKJ2179 | GATATTTCATGCATTTTAAATTTACCGGAGG |
| DOKJ2180 | GagcatgcgacgtcgTTAAAAATTATTCACATTCAAAGCCG |
| DOKJ2181 | GTAGACATGAAATATCTCCTTTTAAATTCAATGTTTCATC |
| DOKJ2182 | GagcatgcgacgtcgCTATTTCCACCCTTTCTCAATC |
| DOKJ2183 | AAATGCATGAAATATCTCCTTTTAAATTCAATGTTTCATC |
| DOKJ2231 | GagcatgcgacgtcgTCATGCTTCGTATCCGACC |
| DOKJ2232 | GATATTTCATGATTAAAAAAATTATTTCTGATTCAGGC |
| DOKJ2233 | TTAATCATGAAATATCTCCTTTTAAATTCAATGTTTCAT |
| DOKJ2277 | TCTCTCATGAAATATCTCCTTTTAAATTCAATGTTTCAT |
| DOKJ2278 | GagcatgcgacgtcgTCAGTCTTCAGGATAAGTTTGTTC |
| DOKJ2279 | ATTTTCATGAAATATCTCCTTTTAAATTCAATGTTTCAT |
| DOKJ2280 | GagcatgcgacgtcgTTAATGTGATTCAATCTTGTATCCGA |
| DOKJ2281 | AACTGCACGAAATATCTCCTTTTAAATTCAATGTTTCAT |
| DOKJ2282 | GagcatgcgacgtcgTTATTCATTCGTCCTTTCTTCAGC |
| DOKJ2283 | GATATTTCATGAGAGATTTAGCAGTTATTGATATC |
| DOKJ2284 | GATATTTCATGAAAATTCTGATTGTTGATGATGAT |
| DOKJ2285 | GATATTTCGTGCAGTTTAAACTCACGACG |
| DOKJ2308 | GagcatgcgacgtcgTCATTCCTGATTTGCCTCCT |
| DOKJ2309 | GATATTTCATGTACGAGGTTGATCGAGA |
| DOKJ2312 | TCGTACATGAAATATCTCCTTTTAAATTCAATGTTTCAT |
| DOKJ2320 | GATATTTCATGATTAAAGCAGAATTCGAGAACA |
| DOKJ2323 | GagcatgcgacgtcgTTAGTTGCCTGGATATTCTGATTC |
| DOKJ2324 | TTAATCATGAAATATCTCCTTTTAAATTCAATGTTTCAT |
| DOKJ2343 | GagcatgcgacgtcgTTATTTTTTCACCACCTTGTATCCA |
| DOKJ2344 | TTGCCCATGAAATATCTCCTTTTAAATTCAATGTTTCAT |
| DOKJ2345 | GATATTTCATGGGCAAGGTATCTGTTTTAAT |

Table S2. Primer sets used for cloning of conjugative expression plasmids.

| Annotation | Locus tag | Primer1 | Primer2 | Primer3 | Primer4 |
| --- | --- | --- | --- | --- | --- |
| response regulator transcription factor | NQ504_01035 | DOKJ2131 | DOKJ2284 | DOKJ2277 | DOKJ2278 |
| ATP-binding protein | NQ504_01040 | DOKJ2131 | DOKJ2285 | DOKJ2281 | DOKJ2282 |
| YhgE/Pip domain-containing protein | NQ504_01050 | DOKJ2131 | DOKJ2320 | DOKJ2324 | DOKJ2323 |
| DegV family protein | NQ504_01055 | DOKJ2131 | DOKJ2232 | DOKJ2233 | DOKJ2231 |
| GntR family transcriptional regulator | NQ504_01100 | DOKJ2131 | DOKJ2178 | DOKJ2181 | DOKJ2180 |
| ROK family protein | NQ504_01140 | DOKJ2131 | DOKJ2283 | DOKJ2277 | DOKJ2278 |
| GntR family transcriptional regulator | NQ504_01165 | DOKJ2131 | DOKJ2179 | DOKJ2183 | DOKJ2182 |
| ATP-binding cassette domain-containing protein | NQ504_01170 | DOKJ2131 | DOKJ2307 | DOKJ2311 | DOKJ2310 |
| FliA/WhiG family RNA polymerase sigma factor | NQ504_08840 | DOKJ2131 | DOKJ2309 | DOKJ2312 | DOKJ2308 |
| response regulator transcription factor | NQ504_01895 | DOKJ2131 | DOKJ2345 | DOKJ2344 | DOKJ2343 |
| sigma-70 family RNA polymerase sigma factor | NQ504_02240 | DOKJ2131 | DOKJ2151 | DOKJ2152 | DOKJ2153 |
| hypothetical protein | NQ504_08745 | DOKJ2131 | DOKJ2154 | DOKJ2155 | DOKJ2156 |

**Movie S1.** Microscopic observation of *L. ruminis* ATCC 25644 during logarithmic phase**.** **(**Magnification**:** ×1000**)** The movie is shown in real time.

<https://doi.org/10.6084/m9.figshare.30469061.v1>

Movie S2. Microscopic observation of *L. ruminis* BKN502 during logarithmic phase. (Magnification: ×1000) The movie is shown in real time.

<https://doi.org/10.6084/m9.figshare.30469091.v1>

Supporting Information, S1. RNA-seq–based gene expression profile of *Ligilactobacillus ruminis* ATCC 25644 wild-type and motile mutant strains. This file contains a comprehensive list of gene expression levels obtained from RNA-seq analysis of wild-type (WT) and motile mutant (Mot) strains of *L. ruminis* ATCC 25644. Each row corresponds to a gene locus, identified by its Locus Tag. For each gene, the following information is included: gene name (if annotated), functional description, transcript length (nt), raw read count, and normalized expression values in FPKM (Fragments Per Kilobase of transcript per Million mapped reads) and TPM (Transcripts Per Million). Data represent values under standard culture conditions and were used for differential expression analysis described in the main text.
